# Morphological and genome-wide evidence for natural hybridisation within the genus *Stipa* (Poaceae)

**DOI:** 10.1038/s41598-020-70582-1

**Published:** 2020-08-14

**Authors:** Evgenii Baiakhmetov, Arkadiusz Nowak, Polina D. Gudkova, Marcin Nobis

**Affiliations:** 1 Institute of Botany, Faculty of Biology, Jagiellonian University, Gronostajowa 3, 30-387 Kraków, Poland; 2 Research Laboratory ‘Herbarium’, National Research Tomsk State University, Lenin 36 Ave, 634050 Tomsk, Russia; 3 Botanical Garden-Centre for Biological Diversity Conservation, Polish Academy of Sciences, Prawdziwka 2, 02-973 Warszawa, Poland; 4 Institute of Biology, Opole University, Oleska 22, 45-052 Opole, Poland; 5 Department of Biology, Altai State University, Lenin 61 Ave, 656049 Barnaul, Russia

**Keywords:** Plant hybridization, Taxonomy, Phylogenetics, Genetic markers, Next-generation sequencing

## Abstract

Hybridisation in the wild between closely related species is a common mechanism of speciation in the plant kingdom and, in particular, in the grass family. Here we explore the potential for natural hybridisation in *Stipa* (one of the largest genera in Poaceae) between genetically distant species at their distribution edges in Mountains of Central Asia using integrative taxonomy. Our research highlights the applicability of classical morphological and genome reduction approaches in studies on wild plant species. The obtained results revealed a new nothospecies, *Stipa* × *lazkovii*, which exhibits intermediate characters to *S. krylovii* and *S. bungeana*. A high-density DArTseq assay disclosed that *S.* × *lazkovii* is an F1 hybrid, and established that the plastid and mitochondrial DNA was inherited from *S. bungeana*. In addition, molecular markers detected a hybridisation event between morphologically and genetically distant species *S. bungeana* and probably *S. glareosa*. Moreover, our findings demonstrated an uncertainty on the taxonomic status of *S. bungeana* that currently belongs to the section *Leiostipa*, but it is genetically closer to *S. breviflora* from the section *Barbatae*. Finally, we noticed a discrepancy between the current molecular data with the previous findings on *S. capillata* and *S. sareptana*.

## Introduction

Hybridisation in the wild between closely related species is a common mechanism of speciation in the plant kingdom^1–7^. Due to the prevalence of polyploidy found in angiosperms it has been estimated that around 11% of flowering plants may have arisen through hybridisation events^4^. In addition, speciation via hybridisation can lead to an equal ploidy number within parental and newly formed species^3^. In general, hybridisation is often accompanied by introgression and causes gene transfer between species via repeated backcrossing^4,8–11^. On the one hand it may have contributed to species diversity and speciation^5,12,13^, on the other, deleterious consequences of hybridisation such as decreased fitness, genetic assimilation and gene swamping may drive populations toward the brink of extinction^14–16^.

In the grass family (Poaceae) hybridisation and introgression are well studied mainly for economically important plants, such as wheats^17,18^, maize^19,20^, rice^21, 22^, barley^23,24^, oats^25,26^, rye^27,28^, sugarcanes^29,30^, and sorghums^31,32^. Nowadays new molecular markers and technologies that first came to the field of agriculture are becoming widely used in studies of wild populations with little or no previous genomic information. For instance, genotyping-by-sequencing (GBS) and GBS-like approaches that were initially developed for maize and barley^33^ help to detect hybridisation and introgression events in many wild plant genera^34–38^.

The genus *Stipa* L. belongs to the subfamily Pooideae and alongside with Bambusoideae (bamboos), and Oryzoideae (rices) form the so-called BOP clade^39^. The BOP species are known as the "cool season" or "pooid" grasses and all are C_3_ and distributed in temperate climates^40^. Following Tzvelev (1974), the genus *Stipa* includes six main sections *Barbatae* Junge, *Leiostipa* Dumort, *Pseudoptilagrostis* Tzvelev, *Regelia* Tzvelev, *Stipa*, and *Smirnovia* Tzvelev^41^, and comprises over 150 species native to Asia, Europe and North Africa^42,43^. In its strict sense, the genus is monophyletic^44,45^, but subdivisions within the genus are not consistently supported by available molecular data^43,46^. Species of the genus are dominants and/or subdominants in steppe plant communities^47–50^, can be used for their classification^51^, and in studies related to climate change^52–54^. Moreover, the species are of great economic importance mainly as pasture and fodder plants, especially in the early phases of development^55^, they can be used for soil remediation processes^56^, and as ornamental plants (e.g. *S. capillata* L., *S. pulcherrima* K. Koch*, S. pennata* L.).

For decades it has been hypothesised that some *Stipa* taxa arose via hybridisation^57–60^. According to our observations, *Stipa* hybrids reproduce vegetatively and, less frequently, sexually^60^. It recently was shown that hybrids in *Stipa* can produce fertile pollen grains and therefore are able to backcross with both parental species^61^. In addition, based on morphology, a hybrid origin can be attributed to ca. 30% of *Stipa* species where only in Middle Asia 23 of 72 species are regarded as nothospecies^43^. For instance, to such taxa belong *S.* × *czerepanovii* Kotukhov (= *S. orientalis* Trin*.* × *S. richteriana* Kar. & Kir.); *S.* × *fallax* M. Nobis & A. Nowak (*S. drobovii* (Tzvel.) Czer. × *S. macroglossa* P. A. Smirn. subsp. *macroglossa*); *S.* × *gegarkunii* P. A. Smirn. (= *S. caucasica* Schmalh*.* × *S. pulcherrima* K. Koch); *S.* × *hissarica* M. Nobis (= *S. lipskyi* Roshev. × *S. orientalis* Trin.); *S.* × *tzveleviana* Kotukhov (= *S. orientalis* × *S. macroglossa* subsp. *kazachstanica*); and *S.* × *zaissanica* Kotukhov (= *S. orientalis* × *S. hohenackeriana* Trin. & Rupr.)^43,60,62,63^.

Heretofore, all putative hybrid taxa within *Stipa* were described based exclusively on morphological comparison. The only exception is *Stipa* × *heptapotamica* Golosk., whose origin has been established using molecular methods^61^. Although its parental species *Stipa richteriana* Kar. & Kir and *S. lessingiana* Trin. & Rupr. were morphologically distant and affiliated to different sections *Leiostipa* and *Subbarbatae* Tzvelev^41,58,64^, genetically they are closely related^65,66^ and able to hybridise with each other^61^.

**Figure 1:**
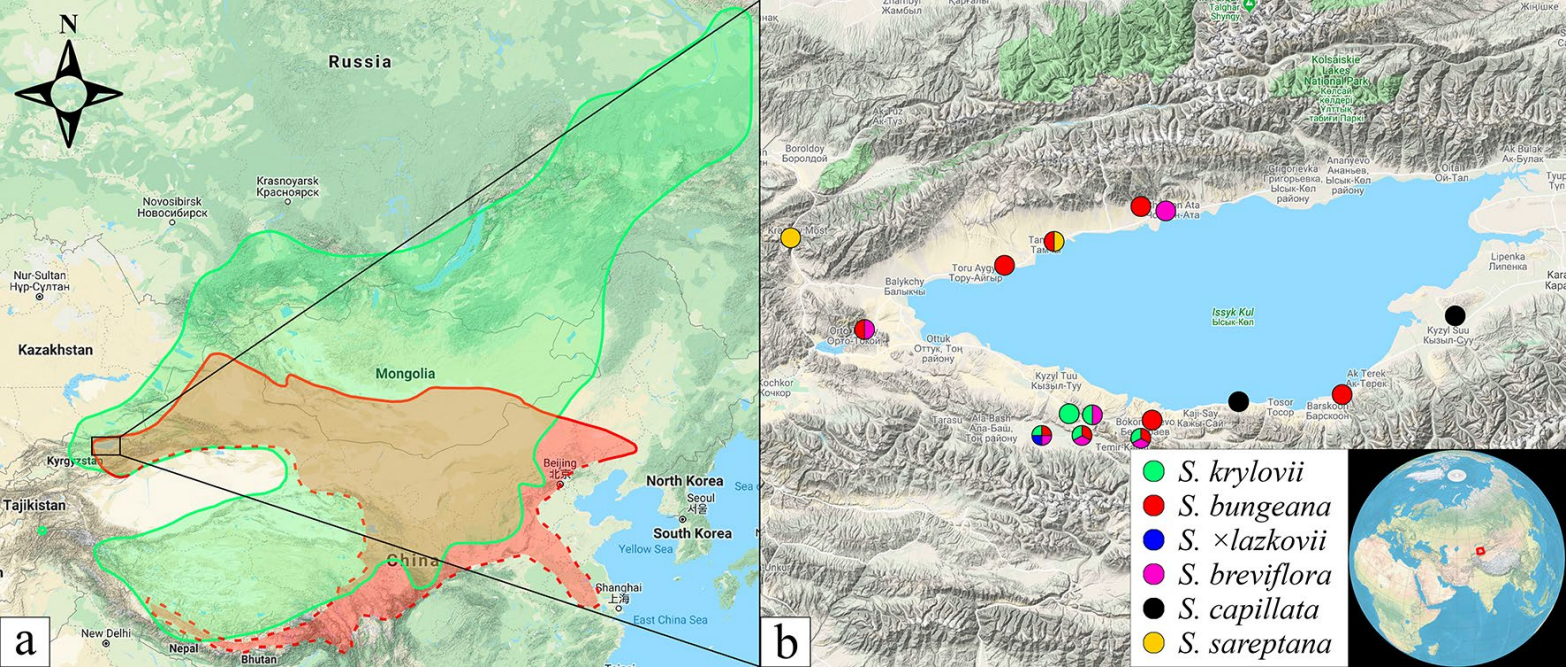
Distribution map represents (**a**) general ranges of *S. krylovii* (green) and *S. bungeana* (red) with the dashed line indicating the hypothetical border, (**b**) localities of the examined specimens used for the molecular analysis. The current map is based on Google Maps.

During field studies in eastern Kyrgyzstan in 2015 and 2017, interesting specimens of *Stipa*, combining characters not observed in the previously described taxa, were found on the south shore of Lake Issyk-Kul (Fig. 1). Due to these specimens seeming to be morphologically intermediate between two species from the same locality, we hypothesised that they can be hybrids between *S. krylovii* Roshev. and *S. bungeana* Trin. Although, traditionally both putative parental taxa were assigned to the section *Leiostipa*^58^, they are distant phylogenetically and belong to two different clades^61,65^. Both of them have wide distribution ranges, *Stipa krylovii* occurs in the Russian Far East and Southern Siberia, Mongolia, China, Northern Nepal, Southern Tajikistan, Eastern Kazakhstan, and Eastern Kyrgyzstan^43,67^, whereas *S. bungeana* is distributed in Southern Mongolia, China, and Eastern Kyrgyzstan^68,69^ (Fig. 1a).

Since hybrids between genetically distant *Stipa* species have not been observed previously in nature, in the current study by using integrative taxonomy based on morphology and high density genome wide genotyping-by-sequencing data, we aim to (1) obtain insight into the extent of hybridisation between *S. krylovii* and *S. bungeana* on macro- and micromorphological levels; (2) assess levels of inter-species gene flow (if present) between the examined *Stipa* taxa; (3) analyse the usefulness of SilicoDArT and SNPs markers for genomic studies in *Stipa*.

## Results

### Numerical analysis

**Figure 2:**
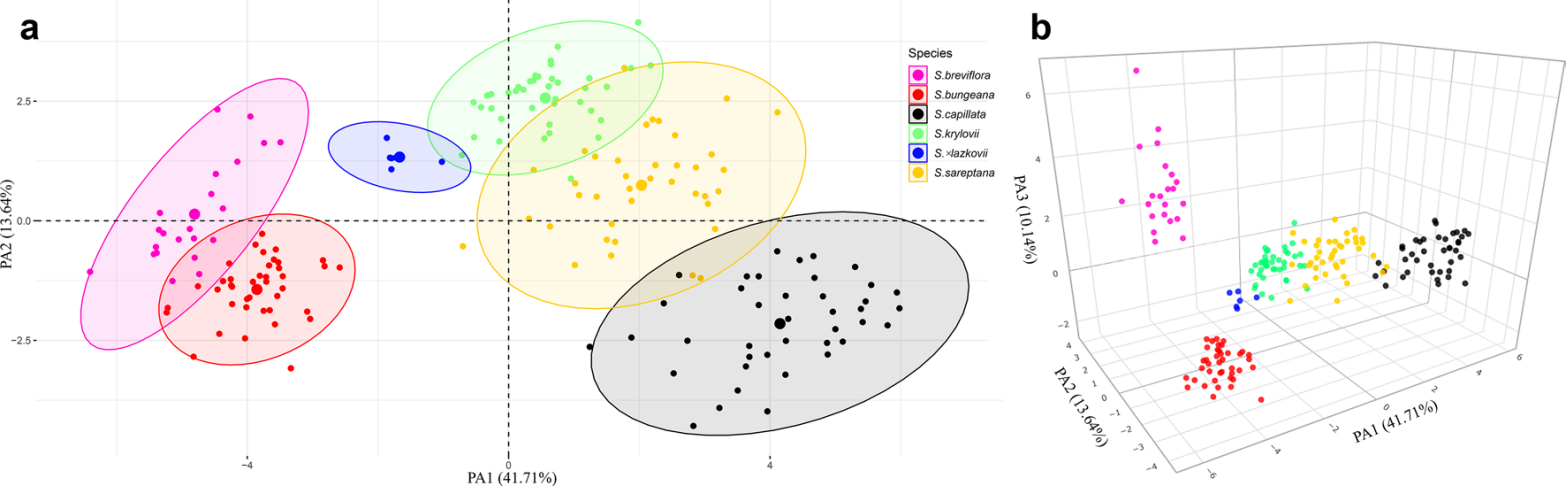
Factor analysis of mixed data performed on 22 quantitative and three qualitative characters of the six examined species of *Stipa*. (**a**) Plot of the two principal axes. (**b**) Plot of the three principal axes. The figure was created using the R-packages factoextra v.1.0.6 (Fig. **a**), https://CRAN.R-project.org/package=factoextra/, and plotly v.4.9.2 (Fig. **b**), https://plotly.com/r/getting-started/.

The factor analysis of mixed data (FAMD) revealed six markedly differentiated groups of OTUs in accordance with the taxonomic classification of the examined taxa (Fig. 2). The first three dimensions explained 41.71%, 13.64%, and 10.14%, of the total variability, respectively. The first dimension is composed, in order of descending contribution, by the quantitative variables AL, Col1L, CL, LG, CvH (Supplementary Table S2, for character abbreviations see Table 1). The second dimension is composed, in order of descending contribution, by the quantitative variables DDL, LHTA, LigIV, WVS, LHD, SL, and the qualitative variable HTTA (Supplementary Table S2). The third dimension is composed, in order of descending contribution, by the quantitative variables HLCol2, HLCol1, WCol1, CBW, and the qualitative variable AdSVL (Supplementary Table S2). The two dimensional plot revealed the overlapping of OTUs belonging to *S. breviflora* and *S. bungeana*, whereas OTUs of *S. sareptana* are slightly overlapped with OTUs of *S. krylovii* and *S. capillata* (Fig. 2a). A clear dispersal of the OTUs could be seen in the three-dimensional plot, where differences between the studied species are explained by the third principal axis (Fig. 2b and in the interactive three-dimensional plot available at https://plot.ly/~eugenebayahmetov/3/). In particular, the third axis differentiates *S. breviflora* and *S. bungeana* as clear non-overlapped clouds of OTUs.

**Table 1:** Morphological characters used in the present study.

Character	Abbreviation
**Quantitative characters (mm)**	
Width of blades of vegetative shoots	WVS
Length of ligules of the middle cauline leaves	LigC
Length of ligules of the internal vegetative shoots	LigIV
Length of lower glume	LG
Length of anthecium	AL
Width of anthecium	AW
Length of callus	CL
Length of hairs on the dorsal part of callus	CdH
Length of hairs on the ventral part of callus	CvH
Length of callus base	CBL
Width of callus base	CBW
Length of hairs on the dorsal line on lemma	LHD
Length of hairs on the ventral line on lemma	LHV
Distance from the end of dorsal line of hairs to the top of lemma	DDL
Distance from the end of ventral line of hairs to the top of lemma	DVL
Length of hairs on the top of lemma	LHTA
Length of lower segment of awn	Col1L
Length of middle segment of awn	Col2L
Length of seta	SL
Length of hairs on lower segment of awn	HLCol1
Length of hairs on middle segment of awn	HLCol2
Width of lower segment of awn	WCol1
**Qualitative characters**	
Character of abaxial surface of vegetative leaves (glabrous, with prickles)	AbSVL
Character of adaxial surface of vegetative leaves (short hairs, long hairs, mixed)	AdSVL
Type of hairs on the top of anthecium (glabrous, poor developed, well developed)	HTTA

In addition, the notch plots of variables showed significant differences between means and the strong evidence of differing medians within all the taxa for CL; AL demonstrates the difference within all the taxa except the pair *S. krylovii* and *S. sareptana*; Col1L exhibits the difference within all the taxa except the pair *S. bungeana* and *S. breviflora*; LG indicates the difference within all the taxa except the pairs *S. capillata* and *S. sareptana*, as well as *S. breviflora* and the putative hybrid (*S. bungeana* × *S. krylovii*), here and below named as *S.* × *lazkovii*; the SL variable shows the difference within all the taxa except the pairs *S.* × *lazkovii* and *S. capillata*, and *S. krylovii* and *S. sareptana* (Supplementary Fig. S1).

Seven notch plots of variables show significant differences between means and the strong evidence of differing medians within *S. bungeana*, *S. krylovii*, and their putative hybrid: AL, CL, Col1L, SL, WCol1, LG, and WVS (Supplementary Fig. S1). At the same time, *S. bungeana*, *S. krylovii* and *S.* × *lazkovii* share six characters that have no significant differences between their means: CvH, CBL, CBW, DVL, HLCol1, and HLCol2. Further, *S.* × *lazkovii* and *S. krylovii* share seven characters with no significant differences between their means, but differ with *S. bungeana*: CdH, DDL, Col2L, LigC, LigIV, AW, LHTA. Finally, only two characters LHD and LHV have no significant differences between means within pairs *S.* × *lazkovii* and *S. krylovii*, and *S.* × *lazkovii* and *S. bungeana*, but have significant differences between means of *S. krylovii* and *S. bungeana* (Supplementary Fig. S1)*.*

### Micromorphology

**Figure 3:**
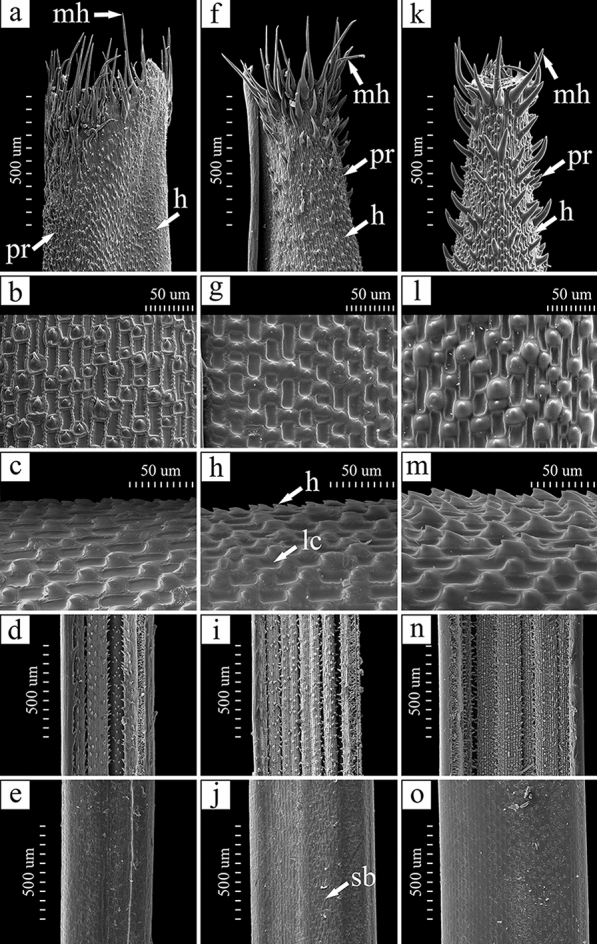
Micromorphological patterns of *Stipa krylovii* (**a-e**), *S.* × *lazkovii* (**f-j**) and *S. bungeana* (**k–o**): top of lemma (**a**, **f**, **k**), lemma abaxial surface (**b-c**, **g-h**, **l-m**), adaxial surface of leaf blade (**d**, **i**, **n**), abaxial surface of leaf blade (**e**, **j**, **o**). Abbreviations: **h** – hooks, **lc** – long cells; **mh** – macrohairs, **pr** – prickles; **sb** – silica bodies.

The micromorphological examination of *Stipa bungeana*, *S. krylovii* and their putative hybrid revealed the pattern of lemma that is typical for the genus *Stipa* (Fig. 3)^45,60,62,70,71^. In all three taxa, the fundamental long cells are rectangular to more or less square in shape. The side walls of long cells are raised and undulate. Silica bodies are sparse or absent, but if present, they are reniform to ovate, whereas cork cells are absent. Hooks are frequent and oriented towards the lemma apex, whereas prickles are present mostly near the lemma apex (Fig. 3). Macrohairs are straight or bent near the base, cylindrical and/or string-like, with a bulbous base and a needle-like apex. They are organised in seven lines. The lemma apex is scabrous due to abundant hooks, prickles and short macrohairs (present especially in *S. bungeana* and in the hybrid), surpassed by a ring of unequal macrohairs. The pattern of lemma apex shows clearly intermediate character of *S.* × *lazkovii* between the two putative parents (Figs. 3a, 3f, 3k).

### DArTseq analysis

**Figure 4:**
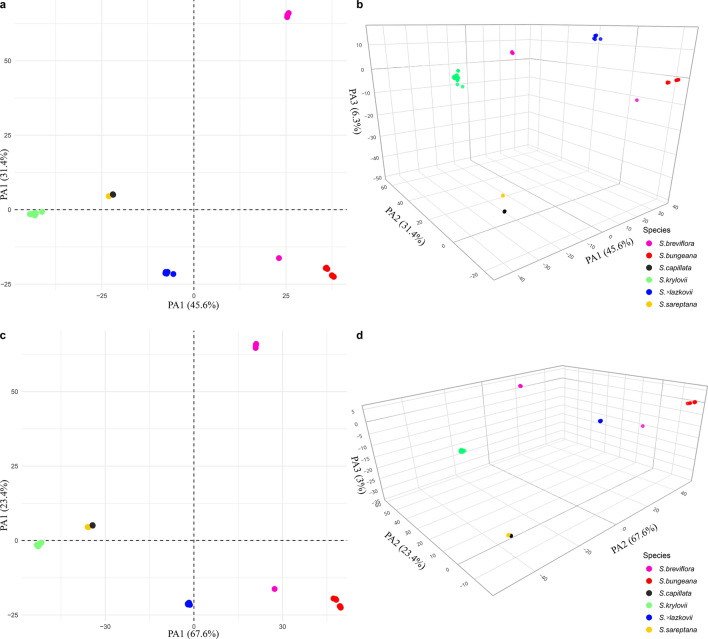
Principal Coordinates Analysis plot based on genetic distances between samples. (**a**) Plot of the two principal axes based on SilicoDArT markers. (**b**) Plot of the three principal axes based on SilicoDArT markers. (**c**) Plot of the two principal axes based on SNPs markers. (**d**) Plot of the three principal axes based on SNPs markers. The figure was created using the R-packages ggplot2 v.3.3.0 (Figs **a** and **c**), https://ggplot2.tidyverse.org/, and plotly v.4.9.2 (Figs **b** and **d**), https://plotly.com/r/getting-started/.

A total of 137,437 SilicoDArT and 125,850 SNPs markers were obtained using a DArTseq high-density assay, of which 76,604 silicoDArT and 19,133 SNPs markers were kept after the filtering steps. The first two axes of principal coordinates analysis (PCoA) explained 77% and 91% of the total genetic divergence within the studied taxa based on the SilicoDArT and SNPs markers, respectively, whereas the third axes explained only 6.3% and 3% (Fig. 4).

In general, based on genetic similarities both markers revealed six markedly differentiated groups (Fig. 4). Most of the specimens are grouped together accordingly to their taxonomical classifications. However, one sample (ID0494394), which morphologically was somewhat similar to *S. breviflora*, is grouping together with *S. bungeana* OTUs and far distant to the rest of OTUs belonging to *S. breviflora*. All *S.* × *lazkovii* specimens have an intermediate position between *S. bungeana* and *S. krylovii*, suggesting an admixed origin. In addition, on the basis of two axes both markers are not allowed to differentiate two taxa, *S. capillata* and *S. sareptana*. On the other hand, the difference can be marked in the three-dimensional plot based on SilicoDArT markers (Figs. 4b, the interactive plot available at https://plot.ly/~eugenebayahmetov/5/), but not in SNPs markers (Fig. 4d, https://plot.ly/~eugenebayahmetov/7/).

**Figure 5:**
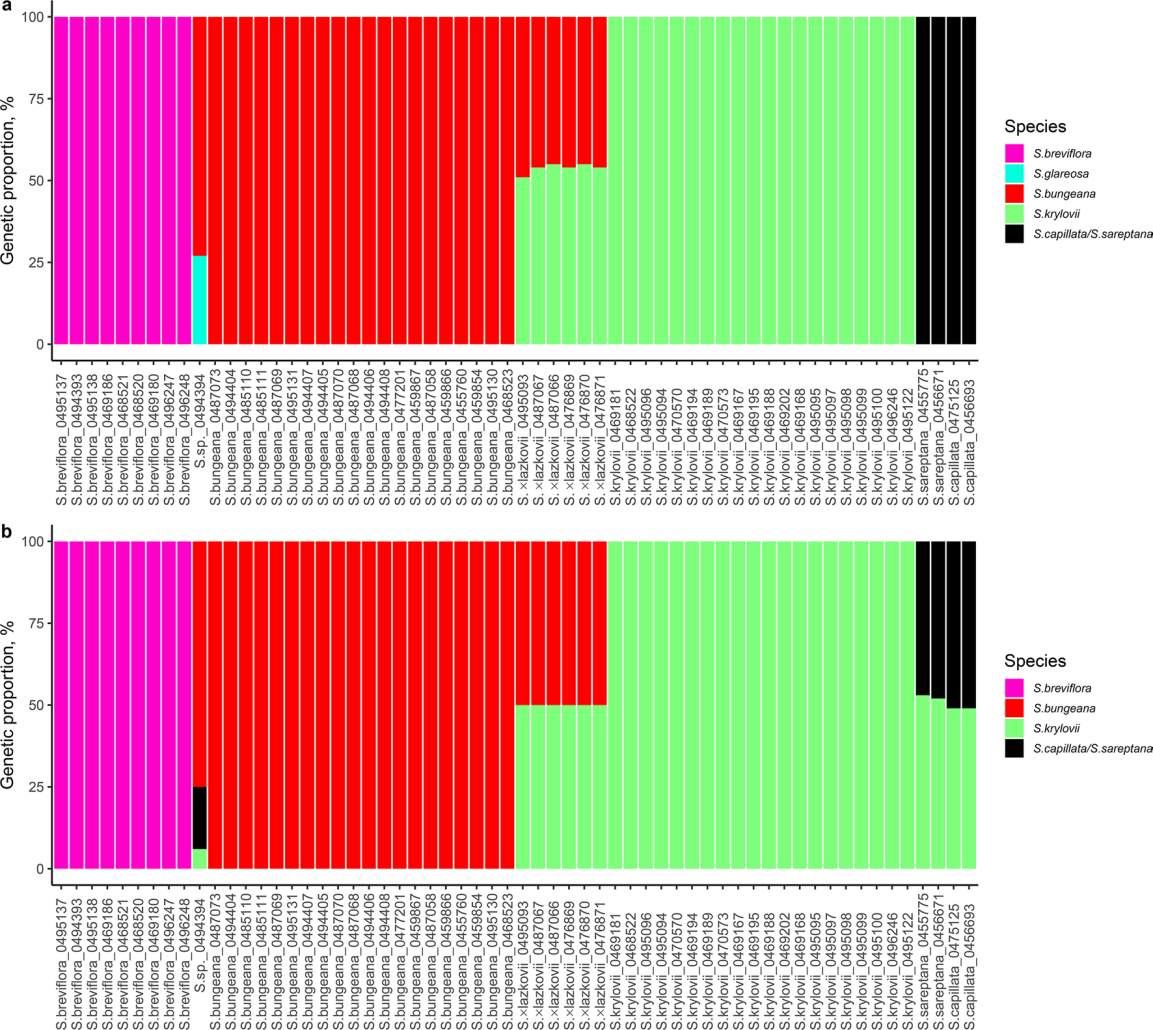
FastSTRUCTURE results based on (**a**) SilicoDArT markers for K = 5 and (**b**) SNPs markers for K = 4. The figure was created using an in-house R script in RStudio v.1.1.463, https://rstudio.com/products/rstudio/.

A fastSTRUCTURE analysis of the SilicoDArT markers revealed the most likely number of clusters at K value of 5 (Fig. 5a). For the SNPs markers, the 'best' K was inferred in fastSTRUCTURE as K = 4 (Fig. 5b). Both analyses defined *S. breviflora*, *S. bungeana*, and *S. krylovii* as clear taxa with the exception of the specimen ID0494394 (Fig. 5) that shares 73% of markers with *S. bungeana* and 27% with probably *S. glareosa*, indicating their first backcross generation progeny (Fig. 5a). The last-mentioned taxon was not present in the analyses, however, it is common in the locality, where the specimen ID0494394 was growing. In case of SNPs markers, the specimen ID0494394 has 75% of markers with *S. bungeana*, 19% with *S. capillata/S. sareptana*, and 6% with *S. krylovii,* suggesting a possible hybridisation between these species followed by backcrossing with *S. bungeana* (Fig. 5b).

The fastSTRUCTURE analyses revealed F1 hybrid specimens between *S. krylovii* and *S. bungeana* due to samples of *S.* × *lazkovii* have admixture between these clusters in a range of 55% and 45% for the SilicoDArT markers (Fig. 5a), and 50/50% for the SNPs markers (Fig. 5b). The fastSTRUCTURE output for the SilicoDArT markers exhibits no difference between *S. capillata* and *S. sareptana* resulting in clustering them together (Fig. 5a), whereas the analysis of the SNPs shows an admixture between *S. capillata/S. sareptana* and *S. krylovii* in a range of 53% and 47%, respectively (Fig. 5b).

**Figure 6:**
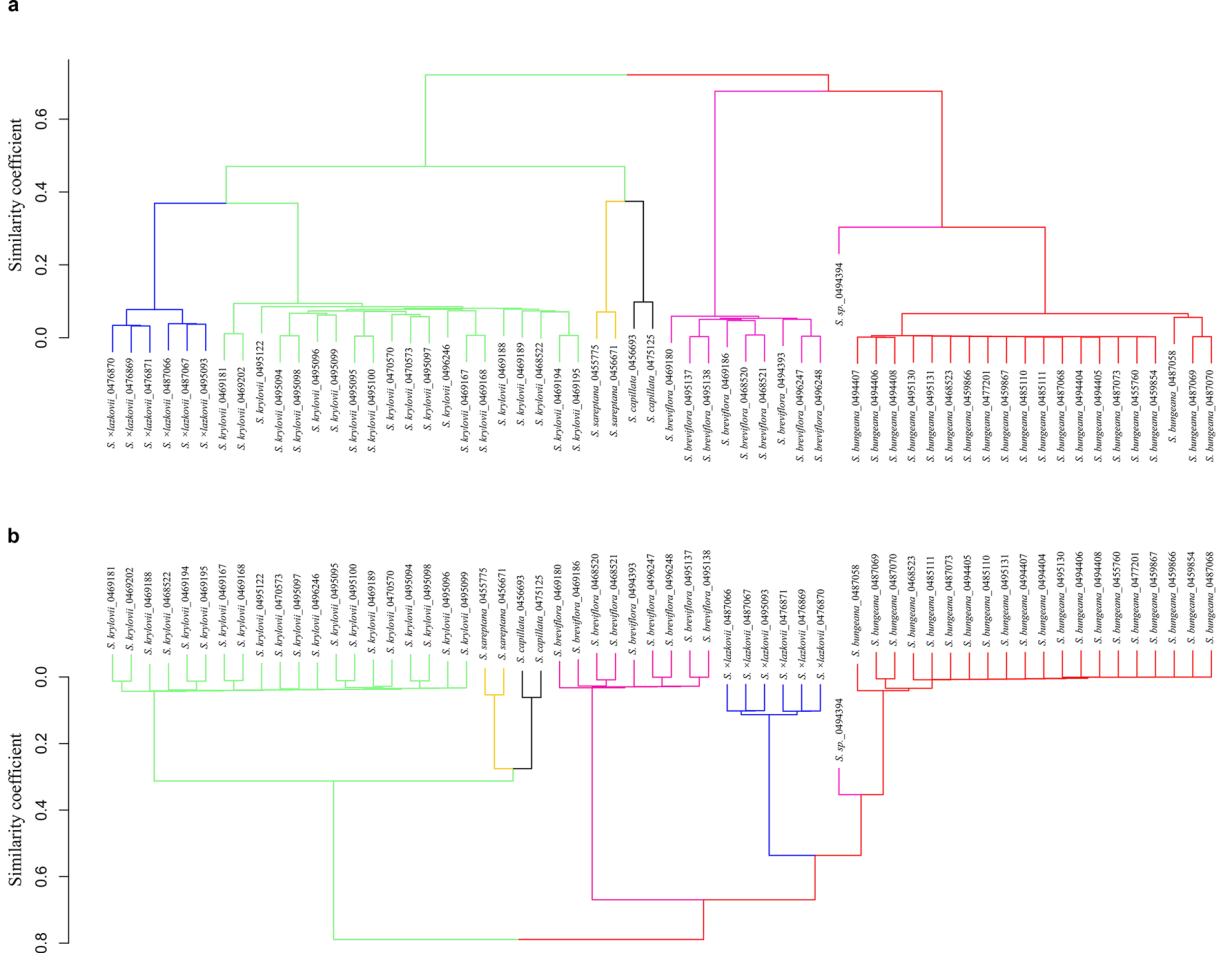
Unweighted Pair Group Method with Arithmetic Mean cluster analyses based on Jaccard's similarity coefficients generated from (**a**) SilicoDArT markers and (**b**) SNPs markers. The figure was created using the R-package stats v.3.6.2, https://www.rdocumentation.org/packages/stats/versions/3.6.2/.

The results of the UPGMA cluster analyses revealed a clear division of samples into two major clades (Fig. 6). According to the clustering obtained with the SilicoDArT markers, the first clade is subdivided into four smaller clusters, specifically, comprising samples of: (1) *S.* × *lazkovii*; (2*) S. krylovii*; (3) *S. sareptana*; (4) *S. capillata* (Fig. 6a). The first two species are genetically closely related to each other and distant to *S. sareptana* and *S. capillata* that together form one sub-cluster. The second clade is composed of three clusters comprising samples of: (1) *S. breviflora*; (2) *S. bungeana*; (3) the sample ID0494394 that is genetically closer to *S. bungeana* than to *S. breviflora*. The UPGMA cluster analysis of the SNPs markers demonstrated the subdivision of samples into the same number of clusters as were obtained for the SilicoDArT markers. However, in this case, specimens of *S.* × *lazkovii* are genetically closer to *S. bungeana*, but not to *S. krylovii*.

**Figure 7:**
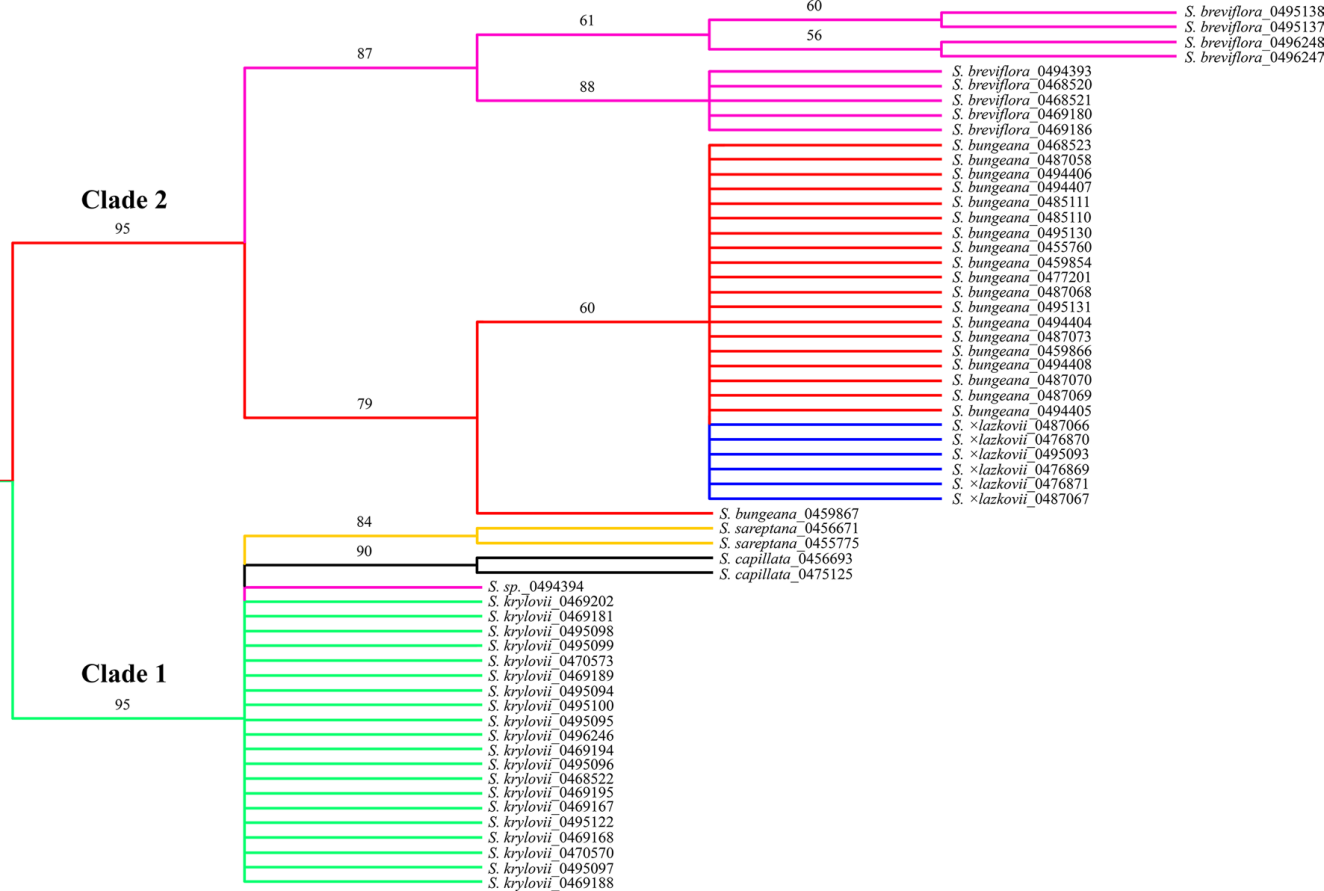
Neighbor-joining tree reconstructed based on the SilicoDArT markers derived from chloroplast and mitochondrial genomes. The bootstrap values > 50% obtained from 10,000 replicates are shown above the branches. The figure was created using Figtree v1.4.4, https://tree.bio.ed.ac.uk/software/figtree/.

Genetic mapping onto chloroplast genomes of *Stipa* species and mitochondria of specimens from the Poaceae family (Supplementary Table S3) revealed 11 SilicoDArT markers assigned to chloroplast DNA and 27 loci assigned to mitochondrial DNA. The downstream neighbour-joining cluster analysis showed grouping of *Stipa* taxa into two main clades (Fig. 7). In the first clade three species could be defined: *S. krylovii*, *S. capillata* (bootstrap support 90%), *S. sareptana* (bootstrap support 84%), with an exception of the specimen ID0494394 that is grouped together with *S. krylovii* (Fig. 7). The second clade comprises a group of *S. bungeana* and *S.* × *lazkovii* (bootstrap support 79%), and the rest of *S. breviflora* specimens with a good bootstrap support of 87% (Fig. 7). All *S.* × *lazkovii* samples are grouping alongside with *S. bungeana*, and one *S. bungeana* specimen (ID0459867) is placed outside the main group of *S. bungeana* and *S.* × *lazkovii* with a bootstrap support of 79% (Fig. 7).

## Discussion

Although many interspecific hybrids have been described in the genus *Stipa*^43,57–59^, so far only a single molecular investigation was performed to verify the origin of one such species, *Stipa* × *heptapotamica*^61^ that appeared to be a hybrid between genetically closely related species^65,66^. The current study is the first report of hybridisation between two genetically distant *Stipa* species, *S. krylovii* and *S. bungeana*^61,65^, at their distribution edges in Mountains of Central Asia (Fig. 1a).

Analyses of morphological variation resulted in a clear delimitation of the studied species (Fig. 2b). Particularly, the main morphological characters (Table 1) show that species *S. capillata*, *S. sareptana*, *S. krylovii*, *S. bungeana*, *S.* × *lazkovii* representing the section *Leiostipa* are quite distant to *S. breviflora* which traditionally has been affiliated to the section *Barbatae*^41^. As expected, the hybrid specimens of *S.* × *lazkovii* were mostly characterised by intermediate morphological traits between the parental taxa *S. krylovii* and *S. bungeana* (Figs. 2 and 3, Supplementary Fig. S1). In addition, some OTUs of *S. sareptana* were slightly overlapped with OTUs of *S. krylovii* and *S. capillata.* However, *S. sareptana* and *S. krylovii* are easy to distinguish based on morphology of leaves (scabrous in *S. sareptana* and glabrous in *S. krylovii*) and the lemma apex (with a poorly developed ring of hairs in *S. sareptana* and with a well-developed ring of hairs in *S. krylovii*)^43,58,67^. As for *S. sareptana* and *S. capillata*, these taxa can be delimited by characteristics of their vegetative leaves (scabrous in *S. sareptana* and glabrous in *S. capillata*) and characters of lemma (hairs on the top in *S. sareptana* and glabrous in *S. capillata*)^43,58,67^.

Both PCoA and fastSTRUCTURE analyses confirmed that *S.* × *lazkovii* is the F1 hybrid of *S. krylovii* and *S. bungeana* (Figs. 4 and 5). In addition, the neighbour-joining cluster analysis identified *S. bungeana* as the source of maternal DNA for all hybrid specimens, suggesting unidirectional hybridisation (Fig. 7). However, due to the small sample size, we cannot exclude either an opposite combination or interspecific gene flow through introgression that could exist in nature, especially since in this area of Issyk-Kul Lake populations of both parental species are extremely large.

Although morphologically *S. bungeana* is considered as a member of the section *Leiostipa*^58^, molecular analyses demonstrated that it is quite distant from *S. krylovii*, *S. capillata*, and *S. sareptana* from the same section (Figs. 4, 6 and 7). These findings support our previous molecular results for these taxa based on a nuclear region^61,65^. In addition, the results of the distance based clustering algorithms UPGMA and NJ revealed that *S. bungeana* is closer to *S. breviflora* then to the rest of *Leiostipa* taxa from the study (Figs. 6 and 7). This result demands further investigations on *S. bungeana* to establish its proper taxonomic place in the genus *Stipa*.

Analyses of molecular markers also revealed that the genetic relationships within some studied taxa are more complex than expected. Firstly, the sample ID0494394, which morphologically was somewhat similar to *S. breviflora*, appeared to be an introgressive hybrid that shares 73% of markers with *S. bungeana* and 27%, more likely, with *S. glareosa* (Fig. 5a)*.* Here, we presume that hybridisation events are happening between *S. bungeana* and *S. glareosa*, because the introgressive hybrid was found on the north shore of Lake Issyk-Kul, where only three *Stipa* taxa were recorded (*S. bungeana*, *S. breviflora*, and *S. glareosa*). However, due to *S. glareosa* was absent in the analyses, a new study focusing on hybridisation should be performed to verify if the gene flow is a common event within these taxa. Secondly, our research demonstrates the discordance between the results of fastStructure and PCoA analyses from one side and the UPGMA and NJ from the other. The first two represent no or almost no difference between *S. capillata* and *S. sareptana* (Figs. 4 and 5). Notwithstanding, the UPGMA and NJ dendrograms show that genetically these taxa can be delimited (Figs. 6 and 7) that supports our previous molecular investigations on these taxa^61,65^. However, in the current research *S. capillata* and *S. sareptana* are grouped together, whereas based on the nuclear Intergenic Spacer (IGS) the last taxon is closer to *S. krylovii*^61,65^. Due to the limited number of analysed specimens in the present and previous studies, we believe that a bigger sample size combining genetics and traditional taxonomy should be undertaken in order to better resolve the relationship between these species.

Until now the DArTseq approach has been used mostly in commercially important plant species^72–78^ and its implication in genomic studies in wild species is still limited^79,80^. Thus, the current study highlights the applicability of genome reduction approaches such as DArTseq in studies on natural hybridisation in wild, and specifically in a grass genus *Stipa*. The high density genome wide genotyping-by-sequencing resulted in a total of 137,437 silicoDArT and 125,850 SNPs markers, of which 76,604 silicoDArT and 19,133 SNPs provided robust information of the *Stipa* genome in the absence of the reference sequence information. Such number of markers is several 100-fold higher than was achieved in our previous study on natural hybridisation in *Stipa*^61^. In particular, by using inter simple sequence repeat markers (ISSR) we were able to detect only 105 polymorphic bands for the *S. heptapotamica* hybrid complex. In addition, dominant markers were used in several genomic studies in *Stipa* and resulted in 372 polymorphic ISSR bands for *S. bungeana*^81^, 34 polymorphic ISSR bands for *S. ucrainica* and *S. zalesskii*^82^, 212 polymorphic ISSR bands for *S. tenacissima*^83^, 231 polymorphic random amplified polymorphic DNA (RAPD) bands for *S. krylovii*^84^, 310 polymorphic RAPD bands for *S. grandis*^85^, and 504 polymorphic sequence-related amplified polymorphism bands for *S. bungeana*^81^. Thus, both silicoDArT and SNPs markers may better suit for genetic diversity studies in *Stipa*. Furthermore, the current study demonstrated the usefulness of silicoDArT markers as a tool to detect chloroplast and mitochondrial loci and thus may help to clarify the maternal inheritance of hybrid species.

### Taxonomic treatment

***Stipa × lazkovii*** M. Nobis & A. Nowak, *nothosp. nov.* (Fig. 3f-j, Supplementary Figs S2 and S3). TYPE: Kyrgyzstan, between Kongurlen and Kultor, 17 km SW from coast of Issyk-Kul, semidesert, N 42°5′47.07′' / E 76°39′6.22′', elev. 1940 m, wp. 930, 6 July 2017, *M. Nobis, E. Klichowska, A. Wróbel, A. Nowak sn*. (holotype KRA 495,093! (specimen in the middle part of the sheet); isotypes KRA 487,067!, 487,066!, 481,608!).

Diagnosis: *Stipa* × *lazkovii* differs from *S. krylovii* Roshev. by having shorter anthecium (7.3–8.5 mm vs. 9.0–11.5), shorter callus (1.8–2.2 vs. 2.3–3.8 mm long), shorter glumes (15–17 vs. 18–28 mm long) as well as by having long prickle-hairs below the top of the anthecium (Fig. 3). Having long prickle-hairs below the top of the anthecium *Stipa* × *lazkovii* is also similar to *S. bungeana*, however differs from it by longer anthecium (7.3–8.5 vs. 4.8–6.0 mm long), longer callus (over 1.8 vs. up to 1.3 mm long), longer glumes (over 15 vs. up to 15 mm long) and narrower leaves (0.5–0.6 vs. 0.6–1.0 mm wide).

Description: Plants perennial, densely tufted, with a few culms and numerous vegetative shoots; culms 35–55 cm tall, 3-noded, glabrous at and below the nodes. Leaves of vegetative shoots: sheaths glabrous, at margins ciliate; ligules truncate, up to 0.2 mm ciliate at margins; blades convolute, up to 25 cm long, 0.5–0.6(–0.7) mm in diameter, adaxial surface densely pubescent with up to 0.1 mm long hairs (prickles), adaxial surface glabrous, rarely very slightly scabrous. Cauline leaves: sheaths glabrous and with white edge, shorter than internodes; ligules 0.5–5 mm long, acute and glabrous; blades glabrous, up to 12 cm long. Panicle up to 25 cm long contracted, at base enclosed by sheath of uppermost leaf, branches erect, setulose, single or paired. Glumes subequal, 15–25 mm long, narrowly lanceolate, tapering into long hyaline apex. Anthecium 7.3–8.5 mm long and 0.7–0.9 mm wide. Callus 1.8–2.2 mm long, densely pilose on ventral and dorsal surfaces, callus base acute, cuneate, scar elliptic. Lemma pale green, on dorsal surface with abundant hooks and with 7 lines of ascending hairs, hairs up to 0.5 mm long, ventral line of hairs terminates at 1.3–1.7 mm below top of lemma and dorsal line terminates at 1.5–2.2 mm below top of lemma; top of lemma scabrous due to hooks and prickles and at apex with a ring of hairs up to 0.5 mm long. Palea equals to lemma in length. Awn 95–118 mm long, bigeniculate; lower segment of column 19–25 mm long, twisted, scabrous due to prickles and short hairs up to 0.15; upper segment of column 11.5–13 mm long, twisted, scabrous due to prickles and short hairs up to 0.2 mm in long; seta flexuous 65–80 mm long, hairs in the lower part of the seta 0.1–0.2 mm long, gradually decreasing in length towards apex. Anthers yellow, 4–5 mm long, glabrous.

Etymology: The name of the taxon honours prof. dr Georgy A. Lazkov (Academy of Sciences, Bishkek, Kyrgyzstan), the eminent botanist, taxonomists and expert of vascular plants of Middle Asian Mountains.

Other specimens studied (paratypes): Kyrgyzstan, western Tian-Shan, Kongurlen Valley, steppe grasslands near the road, 3 km E of Kongurlen settl., to the S of SW part of Issyk-Kul Lake, N 42°5′53.97′' / E 76°38′37.28′', elev. 1945 m, wp. 644, 10 July 2015, *M. Nobis, A. Nowak sn.* (KRA 476,871, 476,870!, 476,869!, WA!).

An identification key to central Asian species of *Stipa* that have scabrous awns or awns that are throughout covered by 0.1–0.3 mm long hairs is given in Supplementary S4.

## Materials and methods

### Plant material

Morphological examination is based on plant specimens deposited in the KRA herbarium (the acronym from Thiers^86^). In total, 188 fully developed *Stipa* samples were studied under a light microscope SMZ800 (Nikon, Japan) including 40 specimens of *S. krylovii*, 40 of *S. bungeana*, 6 of *S.* × *lazkovii*, 22 of *S. breviflora*, 40 of *S. capillata*, and 40 of *S. sareptana*.

For molecular analysis, we collected leaves of plants from localities where *S. krylovii* and *S. bungeana* grow together with their putative hybrid, as well as from areas where *S. krylovii* and *S. bungeana* grow separately from each other (Fig. 1b). Additionally, we included *Stipa* taxa that frequently occur in the area near of Issyk-Kul Lake. In total, we selected 20 specimens of *S. krylovii*, 20 specimens of *S. bungeana*, 6 specimens of *S.* × *lazkovii*, 10 specimens of *S. breviflora*, 2 specimens of *S. capillata*, and 2 specimens of *S. sareptana*. Only one taxon, *S. glareosa*, is not presented in the study due to it was not found in the locality of *S.* × *lazkovii*. Moreover, *S. glareosa* belongs to the section *Smirnovia*^41^ and exhibits unique characters (e.g. long and pilose awns with a single geniculation), which were not observed in any *Stipa* taxa in this region.

All voucher specimens used in the molecular analysis are preserved at KRA (Supplementary Table S1). The names of plants were adopted from the WCSP^87^.

### Macromorphological analyses

For the morphometric analyses, 188 specimens were used as operational taxonomic units (OTUs)^88^. As a first step, the Shapiro–Wilk test was used in the R-package MVN^89^ to assess the normality of the distribution of each character. The non-parametric Spearman's correlation coefficient was used in the R-package MVN to examine relations between the studied characters. The 22 most informative quantitative and three qualitative morphological characters, commonly used in keys and taxonomic descriptions were chosen for the analyses (Table 1).

A Factor Analysis of Mixed Data (FAMD)^90^ was performed in the R-package FactoMineR^91^ to characterise variation within and among groups of taxa without a priori taxonomic classification and to extract the variables that best identified them. The number of principal components included in the analysis was chosen based on Scree’s test^92^. The R-package factoextra^93^ was used to visualise the first two components, whereas the R-package plotly^94^ was chosen to illustrate the first three.

Notch plots were created in the R-package ggplot2^95^ to explore distributional relationships between each response variable and the studied taxa (Supplementary Fig. S1). The notched box plots display a confidence interval around the median, which is normally based on the median ± 1.57 × interquartile range/square root of n. According to this graphical method for data analysis, if the notches of the two boxes do not overlap, there is "strong evidence" (95% confidence) that their medians differ. Additionally, to reveal significant differences between means of particular characters across all examined taxa the nonparametric Kruskal–Wallis test followed by the Wilcoxon rank sum test for post hoc group comparisons were calculated. To address the multiplicity of comparison, the Bonferroni method was applied to calculate corrected p-values.

### Micromorphological examination

The lemma and lamina micromorphology within *Stipa* × *lazkovii*, *S. krylovii*, and *S. bungeana* were examined using scanning electron microscopy (SEM). The dried samples were coated with a gold layer using a Quorum Q150R S coater (Quorum, UK). The SEM images were obtained by a scanning electron microscope S-4700 (Hitachi, Japan). Further, we examined the adaxial and abaxial surfaces of lamina, and five sets of diagnostic characters of lemma micromorphology: (1) long cells, (2) silica bodies, (3) hooks, (4) prickles, (5) macrohairs.

### DNA extraction, amplification, and DArT sequencing

Isolation of genomic DNA was performed from dried leaf tissues using a Genomic Mini AX Plant Kit (A&A Biotechnology, Poland). Quality check, quantification and concentration adjustment for sequencing and genotyping were accomplished using a NanoDrop One (Thermo Scientific, USA) and agarose gel electrophoresis visualisation. The concentration of each sample was adjusted to 50 ng/μL. Purified DNA samples (1 μg for each sample) were sent to Diversity Arrays Technology Pty Ltd (Canberra, Australia) for sequencing and marker identification.

DArTseq represents a combination of a DArT complexity reduction methods and next generation sequencing platforms^96–100^. The technology is optimised for each organism and application in order to select the most appropriate complexity reduction method (both the size of the representation and the fraction of a genome selected for assays). Based on testing several enzyme combinations for complexity reduction Diversity Arrays Technology Pty Ltd selected the PstI-MseI method for *Stipa*.

DNA samples were processed in digestion/ligation reactions as described previously^97^, but replacing a single PstI-compatible adaptor with two different adaptors corresponding to two different Restriction Enzyme (RE) overhangs. The PstI-compatible adapter was designed to include Illumina flowcell attachment sequence, sequencing primer sequence and "staggered", varying length barcode region, similar to the sequence previously reported^33^. Reverse adapter contained flowcell attachment region and MseI-compatible overhang sequence. Only "mixed fragments" (PstI-MseI) were effectively amplified by PCR using an initial denaturation step of 94 °C for 1 min, followed by 30 cycles with the following temperature profile: denaturation at 94 °C for 20 s, annealing at 58 °C for 30 s and extension at 72 °C for 45 s, with an additional final extension at 72 °C for 7 min. After PCR equimolar amounts of amplification products from each sample of the 96-well microtiter plate were bulked and applied to c-Bot (Illumina, USA) bridge PCR followed by sequencing on Hiseq2500 (Illumina, USA). The sequencing (single read) was run for 77 cycles.

Sequences generated from each lane were processed using proprietary DArT analytical pipelines. In the primary pipeline, the fastq files were first processed to filter away poor quality sequences, applying more stringent selection criteria to the barcode region compared to the rest of the sequence. In that way the assignments of the sequences to specific samples carried in the "barcode split" step were very reliable. Approximately 2.5 mln sequences per barcode/sample were identified and used in marker calling.

### DArTseq data analysis

DArTseq produce two types of data: (1) co-dominant single nucleotide polymorphisms (SNPs) markers, and (2) dominant SilicoDArT markers that represent the presence or absence of restriction fragments. All molecular analyses with the DArTseq data (SNPs and SilicoDArT) sets were performed after filtering steps in the R-package dartR^101^ with the following parameters: (1) a scoring reproducibility of 100%, (2) at least 95% loci called (the respective DNA fragment had been identified (= called) in greater than 95% of all individuals), (3) monomorphic loci were removed, (4) SNPs that shared secondaries (had more than one sequence tag represented in the dataset) were randomly filtered out to keep only one random sequence tag.

Three approaches were used to analyse genetic structure of the studied taxa: (1) Principal Coordinates Analysis (PCoA), (2) fastSTRUCTURE analysis, and (3) Unweighted Pair Group Method with Arithmetic Mean (UPGMA). The PCoA analyses based on Euclidean distance matrices were performed using R-packages dartR and visualised by using ggplot2 to show the first two components, and plotly to illustrate the first three components. Genetic structure was then investigated using the fastSTRUCTURE software, which implements the Bayesian clustering algorithm STRUCTURE, assuming Hardy–Weinberg equilibrium between alleles, in a fast and resource-efficient manner^102^. A number of clusters (K-values) ranging from 2 to 10 were tested using the default convergence criterion of 10^−6^ and priors. The most likely K-value was estimated with the best choice function implemented in fastSTRUCTURE. In case of a range of K values, the true K was determined as a value between the estimates predicted by fastSTRUCTURE and based on what made most biological sense. The output matrices for the best K-values were reordered and plotted using an in-house R script in RStudio (Version 1.1.463)^103^. The threshold of 0.10 < q < 0.90 was applied as the most widely utilised measure for the assessment of hybridisation^104–107^. Contributions from each cluster in a range between 45 and 55% were considered as F1 hybrids, while first‐ and second‐generation backcrosses with one parent were considered at values 0.25 and 0.125, respectively^108^. The UPGMA cluster analyses based on Jaccard's distance matrices were performed using R-packages dartR and visualised with stats^109^.

Finally, the SilicoDArT tags were used to determine maternal inheritance of the putative hybrid *Stipa* × *lazkovii*. The trimmed sequences of the parental species *S. krylovii* and *S. bungeana*, and the rest of studied taxa were mapped onto chloroplast genomes of *Stipa* species and mitochondrions of specimens from the Poaceae family (Supplementary Table S3) by using Minimap2^110^. The final binary data matrix was used to generate a neighbor-joining tree (NJ) derived from Jaccard's genetic distances in the fingerprint analysis with missing data software v1.31 (FAMD)^111^ with a set of 10,000 bootstrap replicates. The resulting tree was visualised and edited using Figtree v1.4.4^112^.

## Supplementary information

Supplementary information.

## Data Availability

The datasets used and/or analysed during the current study are available from the corresponding authors upon request.
