# Supplementary material for: Morphological and genome-wide evidence for natural hybridisation within the genus Stipa (Poaceae)
Source: Sci Rep. 2020 Aug 14;10:13803. doi: 10.1038/s41598-020-70582-1 (PMC7427808; doi:10.1038/s41598-020-70582-1)
Supplement: Supplementary file 1 — Supplementary information. [file 41598_2020_70582_MOESM1_ESM.pdf]

# Morphological and genome-wide evidence for natural hybridisation within the genus *Stipa* (Poaceae, sect. *Leiostipa*)

Evgenii Baiakhmetov[1,2*], Arkadiusz Nowak[3,4], Polina D. Gudkova[2,5], Marcin Nobis[1*]

[1] Institute of Botany, Faculty of Biology, Jagiellonian University, Gronostajowa 3, 30-387 Kraków, Poland
[2] Research laboratory 'Herbarium', National Research Tomsk State University, Lenin 36 Ave., 634050 Tomsk, Russia
[3] Botanical Garden-Centre for Biological Diversity Conservation, Polish Academy of Sciences, Prawdziwka 2, 02-973 Warszawa, Poland
[4] Institute of Biology, Opole University, Oleska 22, 45-052 Opole, Poland
[5] Department of Biology, Altai State University, Lenin 61 Ave., 656049 Barnaul, Russia

*evgenii.baiakhmetov@doctoral.uj.edu.pl
m.nobis@uj.edu.pl

Supplementary Table S1. List of samples used for molecular analysis.

| Taxon | Voucher No in KRA | Locality | Latitude | Longitude | Altitude | Data | Collector |
|---|---|---|---|---|---|---|---|
| *S. krylovii* | 0495122 | Kyrgyzstan,<br>SW part of Lake Issyk-Kul,<br>4 km SSW of Bokonbayevo | N 42°4'46.02" | E 76°58'39.87" | 1946 m | 03.07.2018 | M.Nobis,<br>E.Klichowska,<br>A.Wróbel,<br>A.Nowak |
| *S. breviflora*<br>*S. breviflora* | 0468520<br>0468521 | Kyrgyzstan,<br>SW part of Lake Issyk-Kul,<br>2.5 km E of Toguz Bulak | N 42°5'31.57" | E 76°46'21.82" | 2018 m | 10.07.2015 | M.Nobis,<br>A.Nowak |
| *S. krylovii*<br>*S. krylovii*<br>*S. krylovii*<br>*S. krylovii*<br>*S. krylovii*<br>*S. krylovii*<br>*S. krylovii*<br>*S. lazkovii*<br>*S. lazkovii*<br>*S. lazkovii*<br>*S. bungeana*<br>*S. bungeana*<br>*S. bungeana*<br>*S. bungeana*<br>*S. bungeana*<br>*S. bungeana*<br>*S. bungeana*<br>*S. bungeana*<br>*S. bungeana*<br>*S. bungeana*<br>*S. bungeana*<br>*S. bungeana*<br>*S. breviflora*<br>*S. breviflora* | 0495094<br>0495098<br>0495100<br>0495099<br>0495095<br>0495097<br>0495096<br>0495093<br>0487066<br>0487067<br>0487068<br>0487069<br>0487070<br>0477201<br>0494404<br>0494405<br>0494406<br>0494407<br>0494408<br>0495131<br>0485110<br>0485111<br>0495137<br>0495138 | Kyrgyzstan,<br>to the S of SW part of Lake Issyk-Kul,<br>3 km E of Kongurlen settl. | N 42°5'47.07" | E 76°39'6.22" | 1940 m | 06.07.2017 | M.Nobis,<br>E.Klichowska,<br>A.Wróbel,<br>A.Nowak |

| | | | | | | | |
|---|---|---|---|---|---|---|---|
| *S. lazkovii*<br>*S. lazkovii*<br>*S. lazkovii*<br>*S. bungeana* | 0476871<br>0476869<br>0476870<br>0487073 | Kyrgyzstan,<br>to the S of SW part of Lake Issyk-Kul,<br>3 km E of Kongurlen settl. | N 42°5'53.97" | E 76°38'37.28" | 1945 m | 10.07.2015 | M.Nobis,<br>A.Nowak |
| *S. krylovii*<br>*S. bungeana* | 0468522<br>0468523 | Kyrgyzstan,<br>to the S of SW part of Lake Issyk-Kul,<br>3 km NE of Kongurlen settl. | N 42°6'2.04" | E 76°46'35.41" | 2040 m | 10.07.2015 | M.Nobis,<br>A.Nowak |
| *S. krylovii*<br>*S. krylovii* | 0470570<br>0470573 | Kyrgyzstan,<br>to the S of SW part of Lake Issyk-Kul,<br>5 km of Kongurlen settl. | N 42°6'36.66" | E 76°47'18.16" | 2040 m | 10.07.2015 | M.Nobis,<br>A.Nowak |
| *S. bungeana* | 0487058 | Kyrgyzstan,<br>SW part of Lake Issyk-Kul,<br>Bokonbayevo | N 42° 7' 6.49" | E 77° 0' 55.91" | 1799 m | 01.08.2016 | M.Nobis,<br>A.Nobis |
| *S. krylovii*<br>*S. krylovii*<br>*S. breviflora*<br>*S. breviflora* | 0469167<br>0469168<br>0469180<br>0469186 | Kyrgyzstan,<br>SW part of Lake Issyk-Kul,<br>15 km W of Bokonbayevo | N 42°8'10.30" | E 76°48'11.84" | 1900 m | 01.08.2016 | M.Nobis,<br>A.Nobis |
| *S. krylovii*<br>*S. breviflora*<br>*S. breviflora* | 0496246<br>0496247<br>0496248 | Kyrgyzstan,<br>SW part of Lake Issyk-Kul,<br>ca. 15.5 km W of Bokonbayevo | N 42°8'15.86" | E 76°48'18.82" | 1894 m | 04.07.2018 | M.Nobis,<br>E.Klichowska,<br>A.Wróbel,<br>A.Nowak |
| *S. krylovii*<br>*S. krylovii*<br>*S. krylovii*<br>*S. krylovii*<br>*S. krylovii*<br>*S. krylovii* | 0469188<br>0469195<br>0469202<br>0469194<br>0469181<br>0469189 | Kyrgyzstan,<br>SW part of Lake Issyk-Kul,<br>20 km W of Bokonbayevo | N 42°8'26.50" | E 76°45'25.55" | 2030 m | 1.08.2016 | M.Nobis,<br>A.Nobis |
| *S. capillata* | 0475125 | Kyrgyzstan,<br>S part of Lake Issyk-Kul,<br>ca. 20 km W of Barskoon | N 42°10'43.78" | E 77°18'24.06" | 1612 m | 01.08.2016 | M.Nobis,<br>A.Nobis |
| *S. bungeana* | 0459867 | Kyrgyzstan,<br>S part of Lake Issyk-Kul | N 42°11'47" | E 77°39'00" | 1600 m | 16.06.2013 | M.Nobis,<br>A.Nowak |
| *S. bungeana*<br>*S. breviflora* | 0459866<br>0494393 | Kyrgyzstan,<br>15 km W of Lake Issyk-Kul,<br>5 km NE of E part of Orto-Tokoy Reservoir | N 42°21'26" | E 76°03'26" | 1680 m | 16.06.2013 | M.Nobis,<br>A.Nowak |

| | | | | | | | |
|---|---|---|---|---|---|---|---|
| *S. capillata* | 0456693 | Kyrgyzstan,<br>E part of Lake Issyk-Kul,<br>ca. 27 km SWW of Karakol | N 42°23'16.08" | E 78°1'42.76" | 1800 m | 17.06.2013 | M.Nobis,<br>A.Nowak |
| *S. bungeana* | 0459854 | Kyrgyzstan,<br>17 km SW of Lake Issyk-Kul,<br>between Kongurlen and Kultor | N 42°34'14" | E 76°40'18" | 1620 m | 11.06.2013 | M.Nobis,<br>A.Nowak |
| *S. sareptana* | 0455775 | Kyrgyzstan,<br>NW part of Lake Issyk-Kul,<br>Tamchy | N 42°34'28.05" | E 76°41'02.07" | 1625 m | 12.06.2013 | M.Nobis,<br>A.Nowak |
| *S. sareptana* | 0456671 | Kyrgyzstan,<br>35 km W of Lake Issyk-Kul,<br>ca. 1.5 km S of Krasnyy Most | N 42°34'56.07" | E 75°48'25.75" | 1510 m | 16.06.2013 | M.Nobis,<br>A.Nowak |
| *S. bungeana* | 0495130 | Kyrgyzstan,<br>17 km SW of Lake Issyk-Kul,<br>between Kongurlen and Kultor | N 42°37'18" | E 76°59'15" | 1640 m | 14.05.2011 | M.Nobis,<br>A.Nowak |
| *S. breviflora* | 0494394 | Kyrgyzstan,<br>N part of Lake Issyk-Kul,<br>Cholpon-Ata | N 42°38'14" | E 77°03'49" | 1620 m | 14.05.2011 | M.Nobis,<br>A.Nowak |
| *S. bungeana* | 0455760 | Kyrgyzstan,<br>N of Lake Issyk-Kul,<br>7 km NWW of Cholpon-Ata | N 42°39'10.08" | E 76°59'44.48" | 1752 m | 12.06.2013 | M.Nobis,<br>A.Nowak |

Supplementary Table S2. Contribution (%) by dimension of each character in FAMD.

| Character | Dim.1 | Dim.2 | Dim.3 |
|---|---|---|---|
| AL | **8.138459** | 0.25128967 | 0.343414 |
| Col1L | **7.892034** | 0.01385704 | 0.141086 |
| CL | **7.184994** | 0.52827477 | 0.549433 |
| LG | **6.912585** | 1.38953931 | 0.750192 |
| CvH | **5.994953** | 0.0076572 | 0.247512 |
| HTTA | 5.13036 | **9.05892151** | 1.898838 |
| Col2L | 5.095023 | 2.29357402 | 1.600668 |
| CdH | 5.059659 | 2.39238771 | 0.03297 |
| AW | 4.951688 | 1.11235111 | 0.018848 |
| CBL | 4.626069 | 0.80414294 | 2.087042 |
| SL | 4.512454 | **8.00889469** | 0.005742 |
| WCol1 | 4.290667 | 0.83702145 | **10.24068** |
| LigC | 4.10087 | 5.79251874 | 0.33842 |
| CBW | 3.464594 | 0.3523899 | **8.816285** |
| LHTA | 3.265194 | **10.8351853** | 3.145308 |
| LigIV | 2.585415 | **10.0621745** | 0.231904 |
| AdSVL | 2.48356 | 2.8608142 | **11.89666** |
| AbSVL | 2.450702 | 0.0135254 | 0.29022 |
| HLCol2 | 2.358535 | 0.1142349 | **24.57377** |
| HLCol1 | 2.323692 | 0.06843165 | **24.27274** |
| DVL | 2.246823 | 6.478324 | 3.44793 |
| DDL | 1.845006 | **11.965472** | 1.271305 |
| LHD | 1.18748 | **9.47148499** | 0.003277 |
| WVS | 1.141917 | **9.95174283** | 3.469934 |
| LHV | 0.757266 | 5.33579015 | 0.325828 |

Supplementary Table S3. Species names and GenBank accession numbers for the sequences used in this study.

| Taxon | Genetic compartments | Sequence length (bp) | GB accession number |
|---|---|---|---|
| *Stipa richteriana* | Chloroplast | 137 831 | MG052612.1 |
| *Stipa lipskyi* | Chloroplast | 137 854 | KT692644.1 |
| *Stipa purpurea* | Chloroplast | 137 370 | NC_029390.1 |
| *Stipa ovczinnikovii* | Chloroplast | 137 874 | NC_037034.1 |
| *Stipa jagnobica* | Chloroplast | 137 827 | NC_037029.1 |
| *Stipa hohenackeriana* | Chloroplast | 137 753 | NC_037028.1 |
| *Stipa narynica* | Chloroplast | 137 854 | NC_037032.1 |
| *Stipa magnifica* | Chloroplast | 137 848 | NC_037031.1 |
| *Stipa arabica* | Chloroplast | 137 757 | NC_037024.1 |
| *Stipa orientalis* | Chloroplast | 137 822 | NC_037033.1 |
| *Stipa lessingiana* | Chloroplast | 137 829 | NC_037030.1 |
| *Stipa caucasica* | Chloroplast | 137 798 | NC_037027.1 |
| *Stipa capillata* | Chloroplast | 137 830 | NC_037026.1 |
| *Stipa borysthenica* | Chloroplast | 137 825 | NC_037025.1 |
| *Stipa zalesskii* | Chloroplast | 137 836 | NC_037037.1 |
| *Tripsacum dactyloides* | Mitochondrion | 704 100 | NC_008362.1 |
| *Hordeum vulgare* | Mitochondrion | 525 599 | AP017300.1 |
| *Zea mays* | Mitochondrion | 569 630 | NC_007982.1 |
| *Triticum aestivum* | Mitochondrion | 452 526 | MH051716.1 |
| *Eleusine indica* | Mitochondrion | 520 691 | NC_040989.1 |
| *Sorghum bicolor* | Mitochondrion | 468 628 | NC_008360.1 |
| *Oryza sativa* | Mitochondrion | 637 692 | JF281153.1 |
| *Aegilops speltoides* | Mitochondrion | 476 091 | NC_022666.1 |
| *Alloteropsis semialata* | Mitochondrion | 442 063 | MH644808.1 |
| *Lolium perenne* | Mitochondrion | 678 580 | JX999996.1 |
| *Saccharum officinarum* | Mitochondrion | 300 784 | NC_031164.1 |
| *Saccharum officinarum* | Mitochondrion | 144 698 | LC107875.1 |

Supplementary S4. Identification key to central Asian species of *Stipa* that have scabrous awns or awns that are throughout covered by 0.1–0.3 mm long hairs (shorter than a diameter of the awn).

1. Glumes 9–15 mm long, callus 0.5–1.3 mm long, anthecium 5–7 mm long........................................................**2**

– Glumes 15–35 mm long, callus 1.5–4 mm long, anthecium 7–14 mm long ......................................................**3**

2. Callus 1–1.3 mm long, awn with hairs up to 0.1 mm long, ligules of vegetative shoots 0.2–0.5 mm long, lemma with 7 lines of hairs, of which the dorsal one terminates below the half of the lemma length ........................................................................................................................................ ***S. bungeana*** Trin.

– Callus (0.5–)0.6–0.8(–1) mm long, awn with hairs 0.2–0.3 mm long, ligules of vegetative shoots up to 0.2 mm long densely hairy on margins, lemma with indistinct lines of hairs or all-around pilose, hairs terminate in the upper half of the lemma length ................................ ***S. richteriana*** subsp. ***jagnobica*** (Ovcz. & Czuk.) Tzvelev

3. Anthecium with well developed, dense ring of hairs at the apex ........................................................................**4**

– Anthecium without or with poorly developed ring of hairs at the apex ................................................................**8**

4. Abaxial (lower) surface of blades of vegetative leaves usually glabrous, rarely slightly scabrous, adaxial (upper) surface densely covered with hairs up to 0.1 mm long .......................................................................................**5**

– Abaxial surface of blades of vegetative leaves usually scabrous or glabrous, adaxial surface densely covered with hairs 0.2–0.5 mm long, or a mixture of shorter and longer hairs ................................................................**7**

5. Ligules of vegetative leaves 0.3–2 mm long, callus 1.5–2 mm long ......................... ***S. margelanica*** P. A. Smirn.

– Ligules of vegetative leaves up to 0.2 mm long ....................……………………………………………........**6**

6. Callus (2.1–)2.3–3.8(–4.1) mm long, anthecium 9.0–11.5 mm long, glumes 18–28 mm long .............
………………………………………………………………………………………..……***S. krylovii*** Roshev.

– Callus 1.8–2.2 mm long, anthecium 7.3–8.5 mm, glumes 15–17 .................. ***S.*** **×** ***lazkovii*** M. Nobis & A. Nowak

7. Column 5–9(–10) cm long, anthecium 14–16.5(–17.5) mm, and awn (18–)22–27(–30) cm, middle strip of hairs on the lemma extends up to its lower 1/4–1/2 length, abaxial surface of leaves glabrous and smooth ................................................................................................................................ ***S. grandis*** P. A. Smirn.

- Column less than 3–4.5 cm long, anthecium 13–14 mm, awn 13–17 cm, middle strip of hairs on the lemma extends above its 1/2 length, abaxial surface of leaves scabrous or glabrous .................. ***S. baicalensis*** Roshev.

8. Lemma with poorly developed ring of hairs at the apex, abaxial surface of leaves of vegetative shoots scabrous due to prickles and spinules and adaxial covered with a mix of short and long hairs (long hairs present only on marginal ribs) ................................................................................................ ***S. sareptana*** A. K. Becker

– Lemma glabrous at the apex or rarely with scattered hairs and/or prickles near the lemma margins, abaxial surface of leaves of vegetative shoots scabrous to almost glabrous and adaxial densely covered with 0.2–0.5 mm long hairs (rarely, long hairs are present only on marginal ribs) .................................................................**9**

9. Anthecium 7–11 mm long, awn 7–11 cm long, internodes usually longer than culm sheaths ………..
................................................................................................................................ ***S. karakabinica*** Kotukhov

– Anthecium (10–)11–13(–14) mm long, awn (10–)12–22 cm long, internodes usually shorter than culm sheaths ................................................................................................................................ ***S. capillata*** L

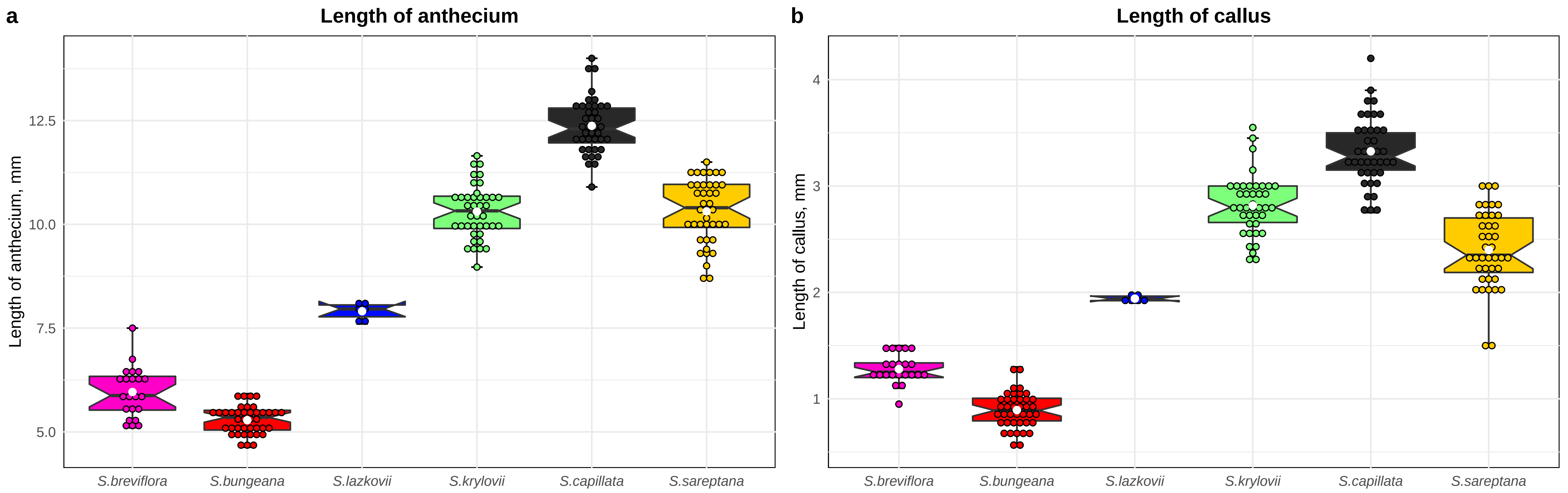


| | *S.breviflora* | *S.bungeana* | *S.lazkovii* | *S.krylovii* | *S.capillata* |
|---|---|---|---|---|---|
| *S.bungeana* | *** | | | | |
| *S.lazkovii* | ** | ** | | | |
| *S.krylovii* | *** | *** | ** | | |
| *S.capillata* | *** | *** | ** | *** | |
| *S.sareptana* | *** | *** | ** | | *** |

| | *S.breviflora* | *S.bungeana* | *S.lazkovii* | *S.krylovii* | *S.capillata* |
|---|---|---|---|---|---|
| *S.bungeana* | *** | | | | |
| *S.lazkovii* | ** | ** | | | |
| *S.krylovii* | *** | *** | ** | | |
| *S.capillata* | *** | *** | ** | *** | |
| *S.sareptana* | *** | *** | ** | *** | *** |

c

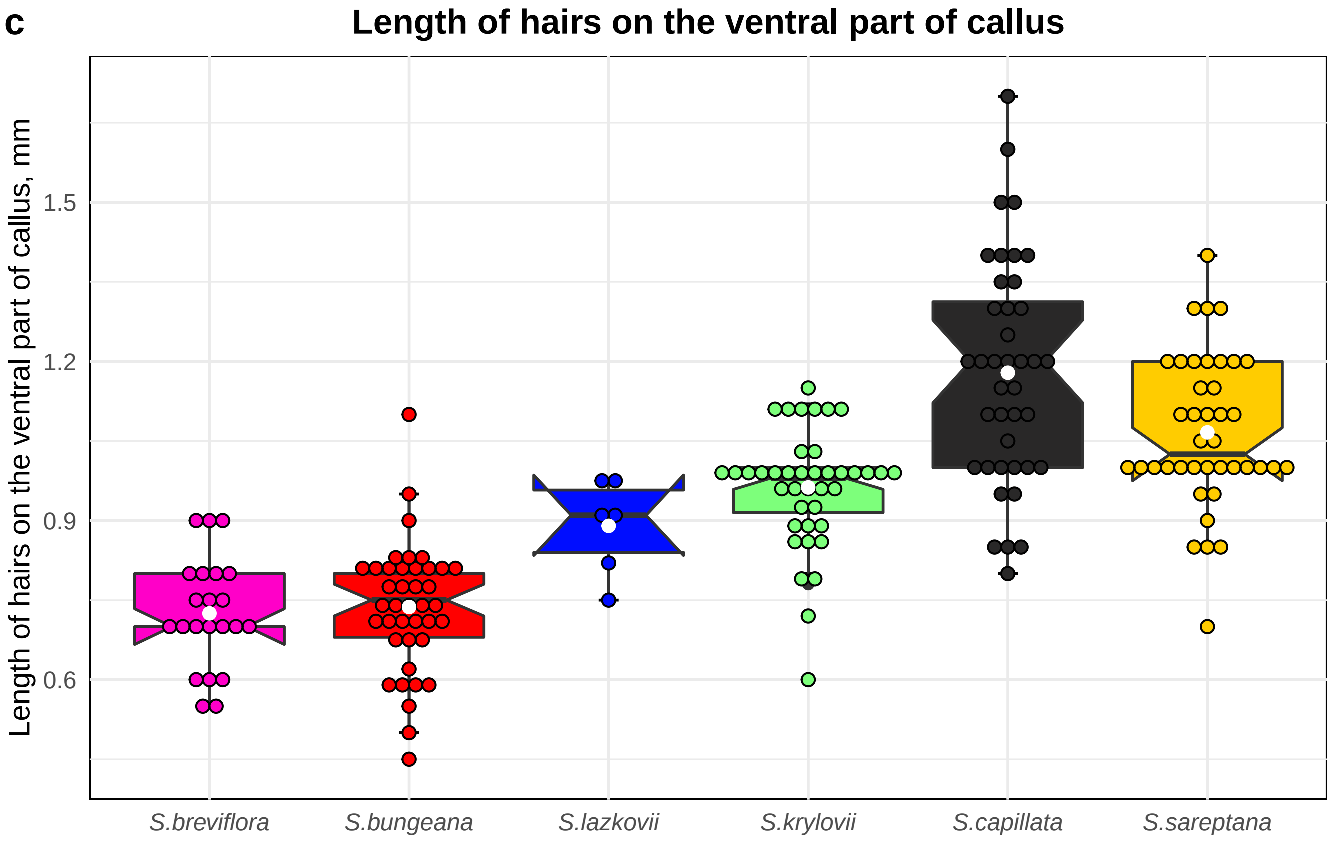


| | S.breviflora | S.bungeana | S.lazkovii | S.krylovii | S.capillata |
|---|---|---|---|---|---|
| S.bungeana | | | | | |
| S.lazkovii | * | . | | | |
| S.krylovii | *** | *** | | | |
| S.capillata | *** | *** | * | *** | |
| S.sareptana | *** | *** | * | ** | |

d

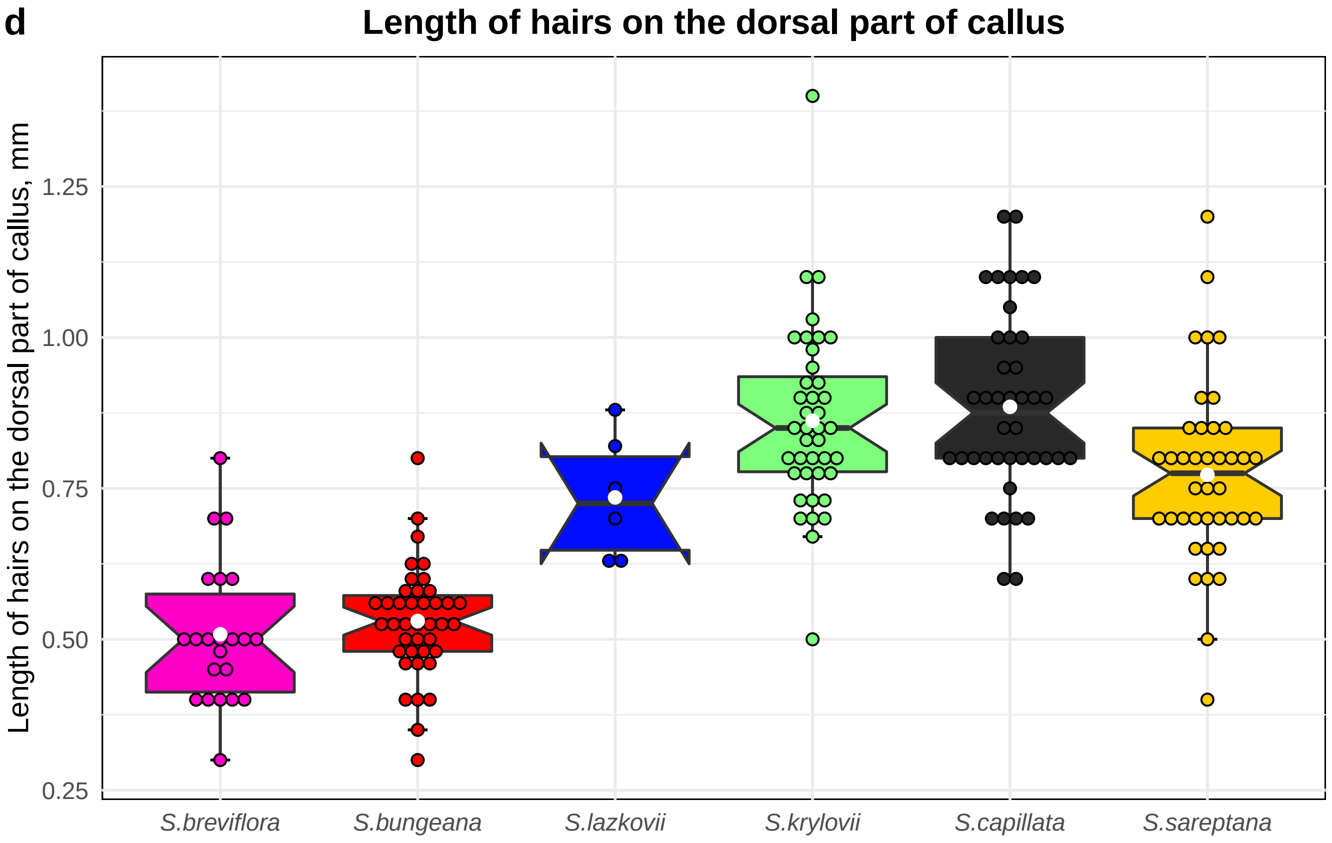


| | S.breviflora | S.bungeana | S.lazkovii | S.krylovii | S.capillata |
|---|---|---|---|---|---|
| S.bungeana | | | | | |
| S.lazkovii | * | ** | | | |
| S.krylovii | *** | *** | | | |
| S.capillata | *** | *** | | | |
| S.sareptana | *** | *** | | . | * |

e

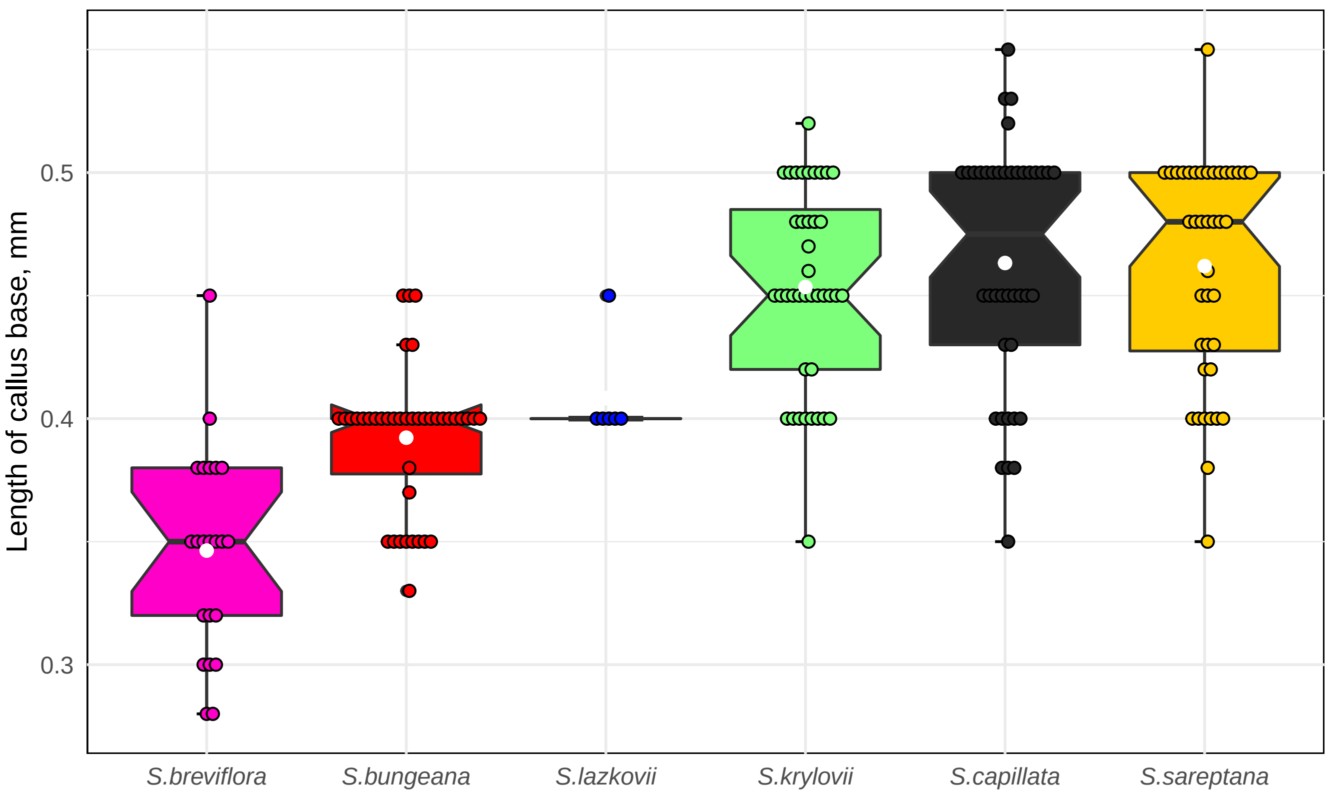


| | S.breviflora | S.bungeana | S.lazkovii | S.krylovii | S.capillata |
|---|---|---|---|---|---|
| S.bungeana | *** | | | | |
| S.lazkovii | * | | | | |
| S.krylovii | *** | *** | | | |
| S.capillata | *** | *** | | | |
| S.sareptana | *** | *** | | | |

f

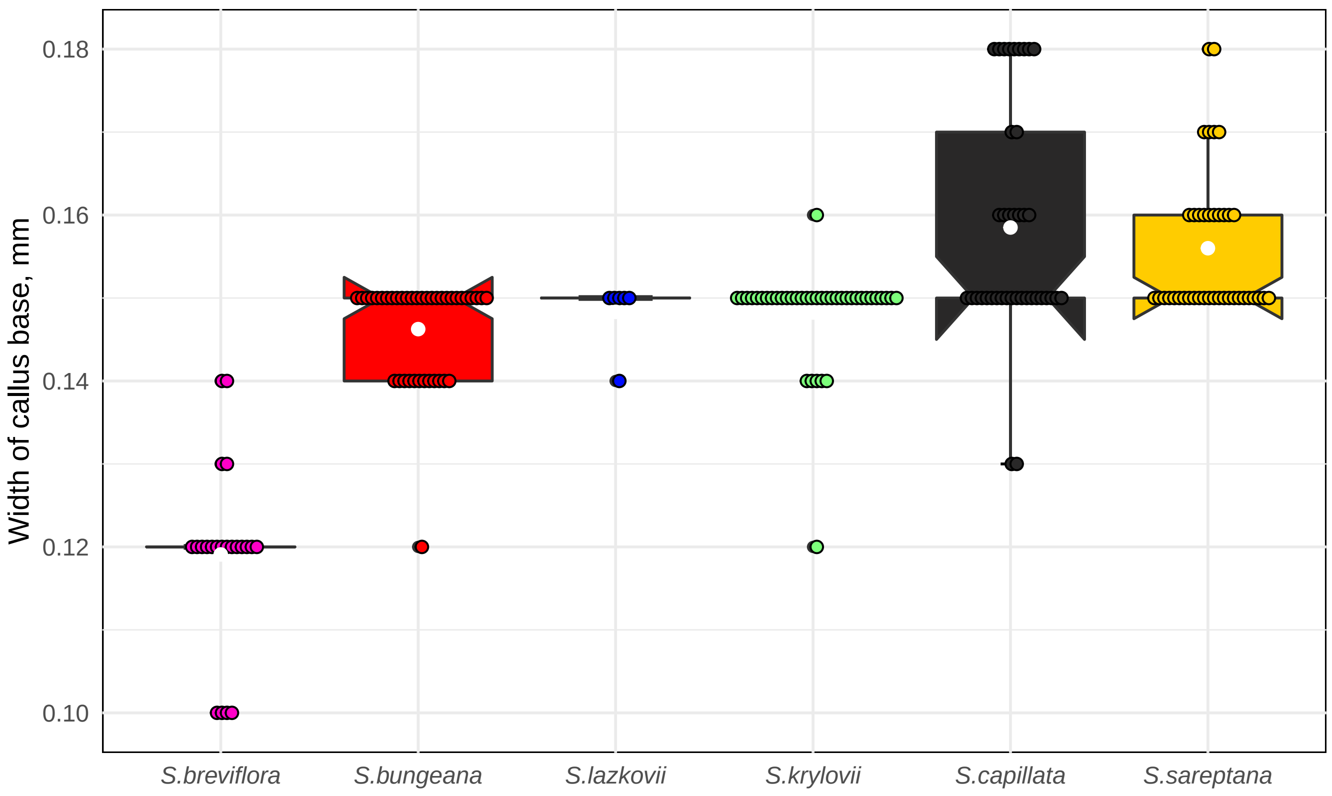


| | S.breviflora | S.bungeana | S.lazkovii | S.krylovii | S.capillata |
|---|---|---|---|---|---|
| S.bungeana | *** | | | | |
| S.lazkovii | ** | | | | |
| S.krylovii | *** | | | | |
| S.capillata | *** | *** | | *** | |
| S.sareptana | *** | *** | | *** | |

g

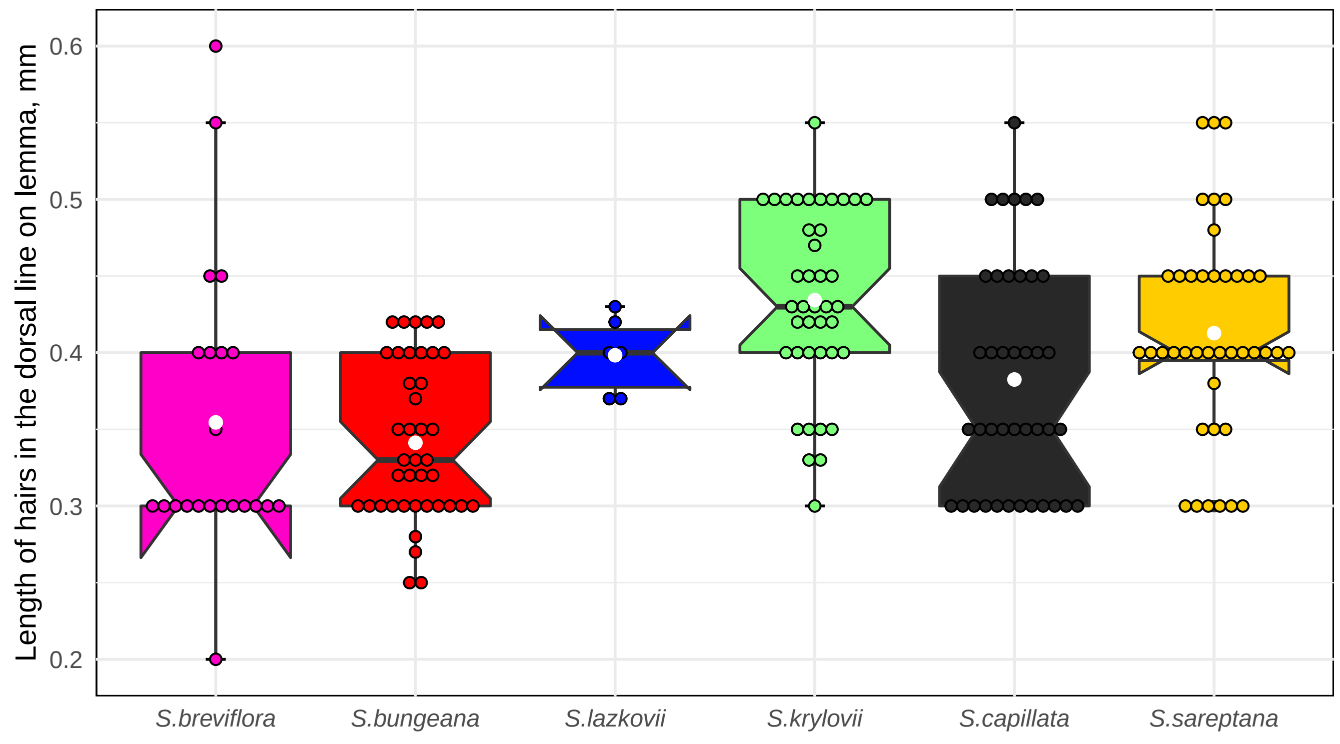


h

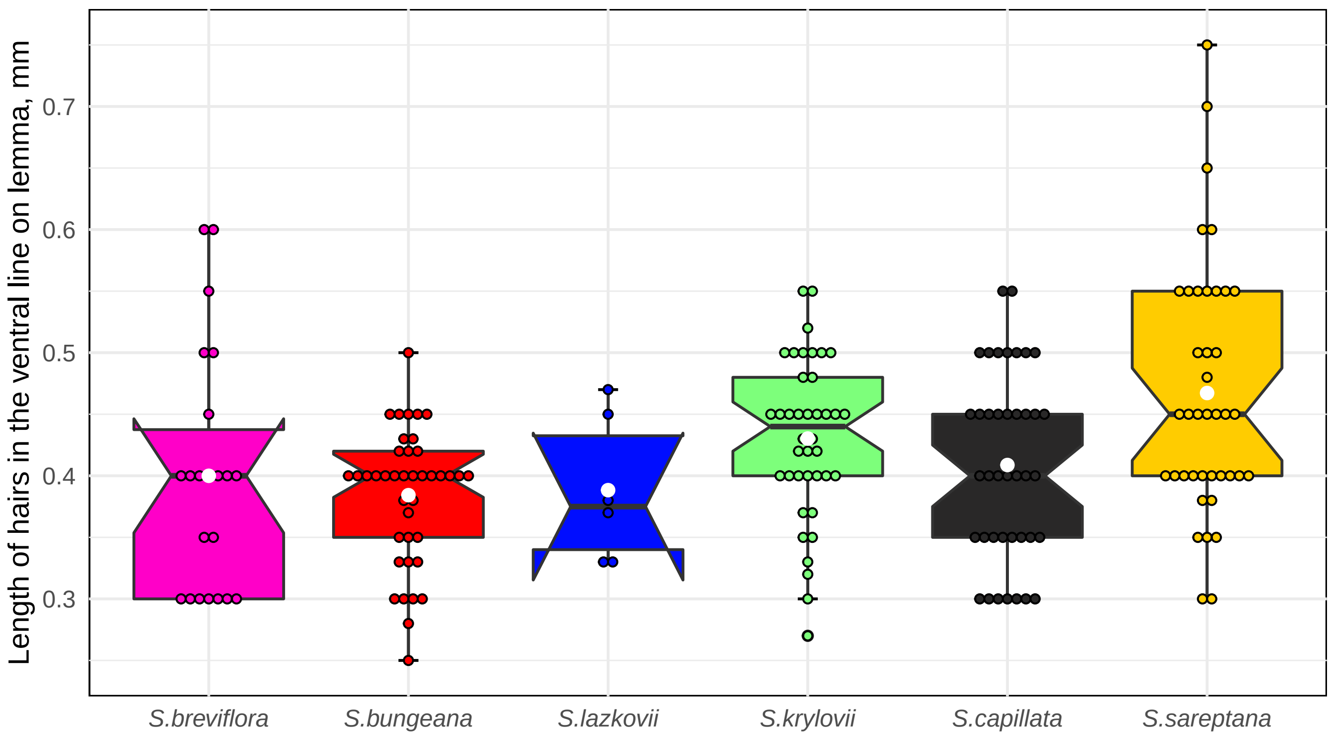


| | *S.breviflora* | *S.bungeana* | *S.lazkovii* | *S.krylovii* | *S.capillata* |
|---|---|---|---|---|---|
| *S.bungeana* | | | | | |
| *S.lazkovii* | | | | | |
| *S.krylovii* | ** | *** | | | |
| *S.capillata* | | | | * | |
| *S.sareptana* | . | *** | | | |

| | *S.breviflora* | *S.bungeana* | *S.lazkovii* | *S.krylovii* | *S.capillata* |
|---|---|---|---|---|---|
| *S.bungeana* | | | | | |
| *S.lazkovii* | | | | | |
| *S.krylovii* | | * | | | |
| *S.capillata* | | | | | |
| *S.sareptana* | | ** | | | |

i

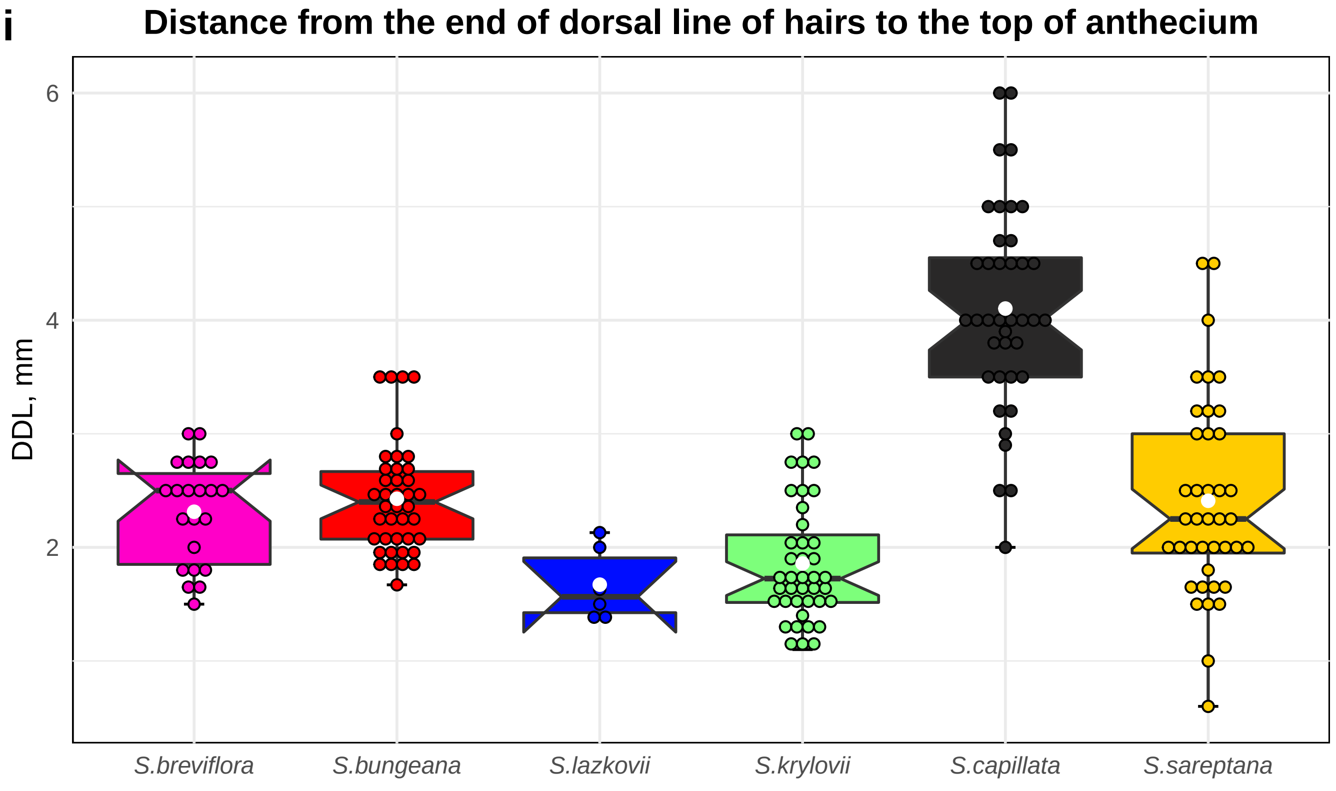


j

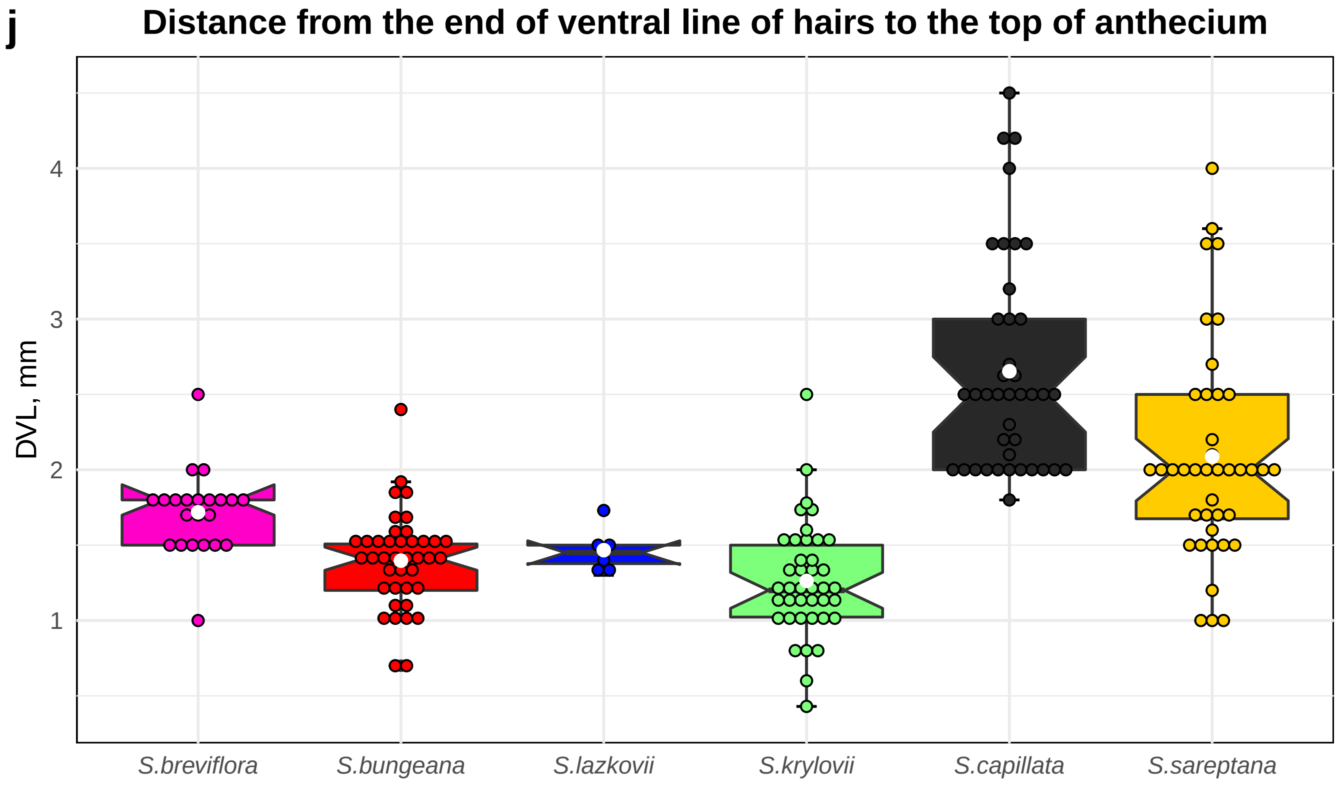


| | *S.breviflora* | *S.bungeana* | *S.lazkovii* | *S.krylovii* | *S.capillata* |
|---|---|---|---|---|---|
| *S.bungeana* | | | | | |
| *S.lazkovii* | . | * | | | |
| *S.krylovii* | * | *** | | | |
| *S.capillata* | *** | *** | ** | *** | |
| *S.sareptana* | | | | * | *** |

| | *S.breviflora* | *S.bungeana* | *S.lazkovii* | *S.krylovii* | *S.capillata* |
|---|---|---|---|---|---|
| *S.bungeana* | *** | | | | |
| *S.lazkovii* | | | | | |
| *S.krylovii* | *** | | | | |
| *S.capillata* | *** | *** | ** | *** | |
| *S.sareptana* | | *** | | *** | ** |

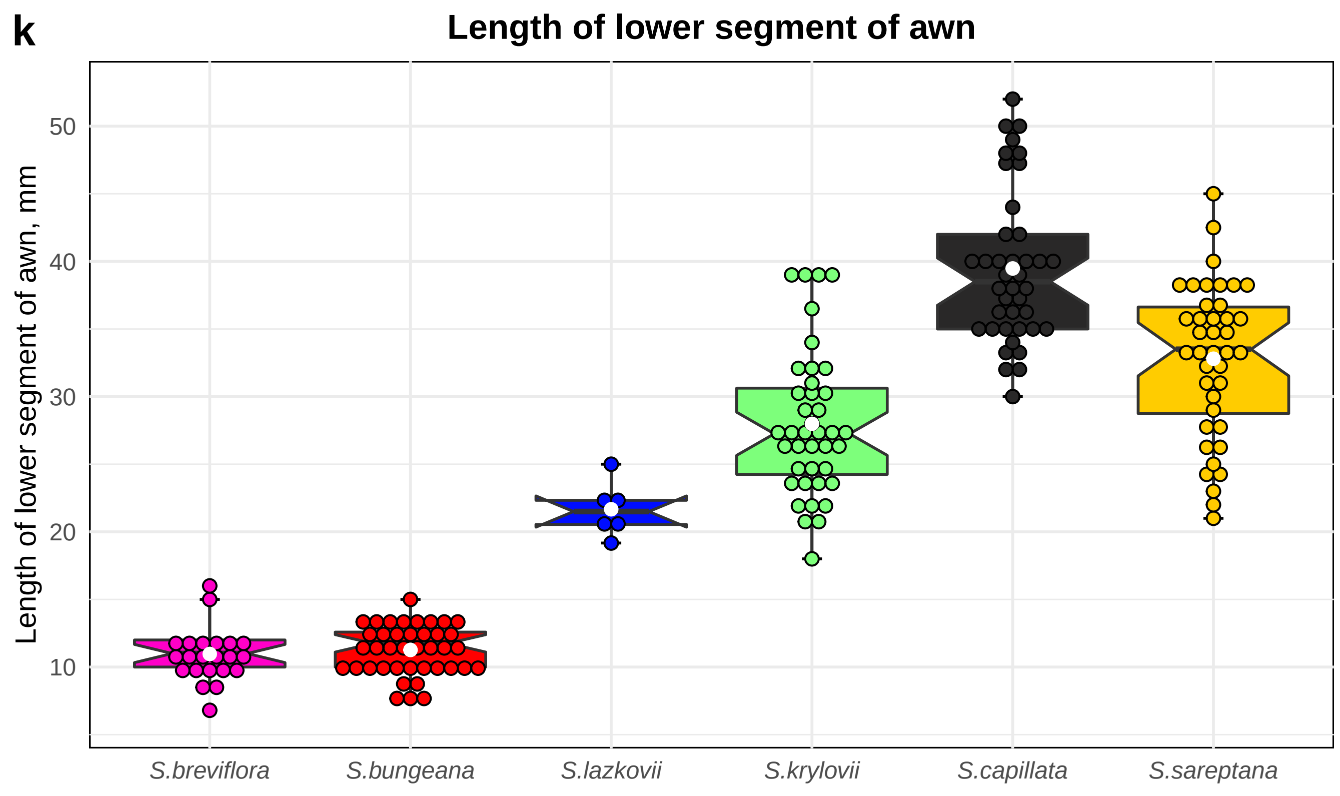


| | S.breviflora | S.bungeana | S.lazkovii | S.krylovii | S.capillata |
|---|---|---|---|---|---|
| S.bungeana | | | | | |
| S.lazkovii | ** | ** | | | |
| S.krylovii | *** | *** | * | | |
| S.capillata | *** | *** | ** | *** | |
| S.sareptana | *** | *** | ** | ** | *** |

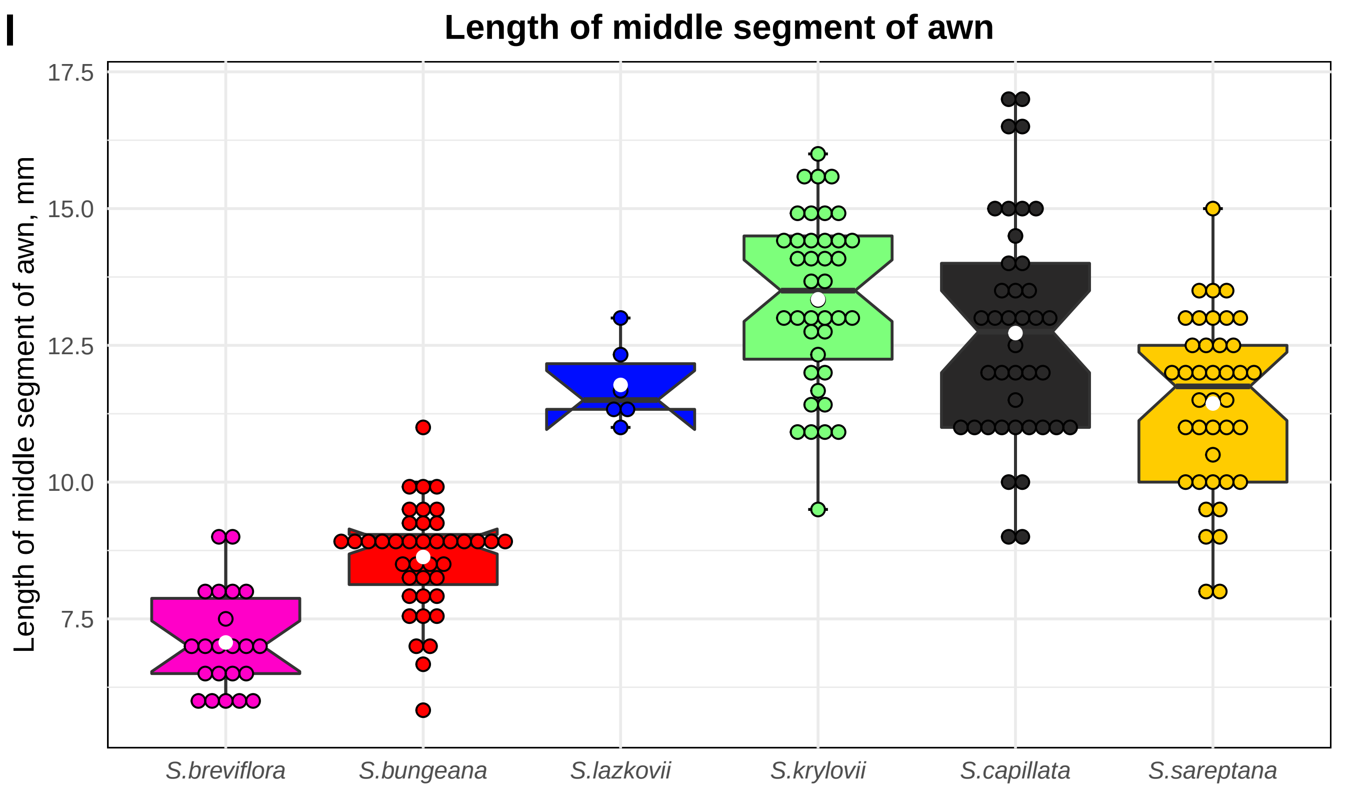


| | S.breviflora | S.bungeana | S.lazkovii | S.krylovii | S.capillata |
|---|---|---|---|---|---|
| S.bungeana | *** | | | | |
| S.lazkovii | ** | ** | | | |
| S.krylovii | *** | *** | | | |
| S.capillata | *** | *** | | | |
| S.sareptana | *** | *** | | *** | |

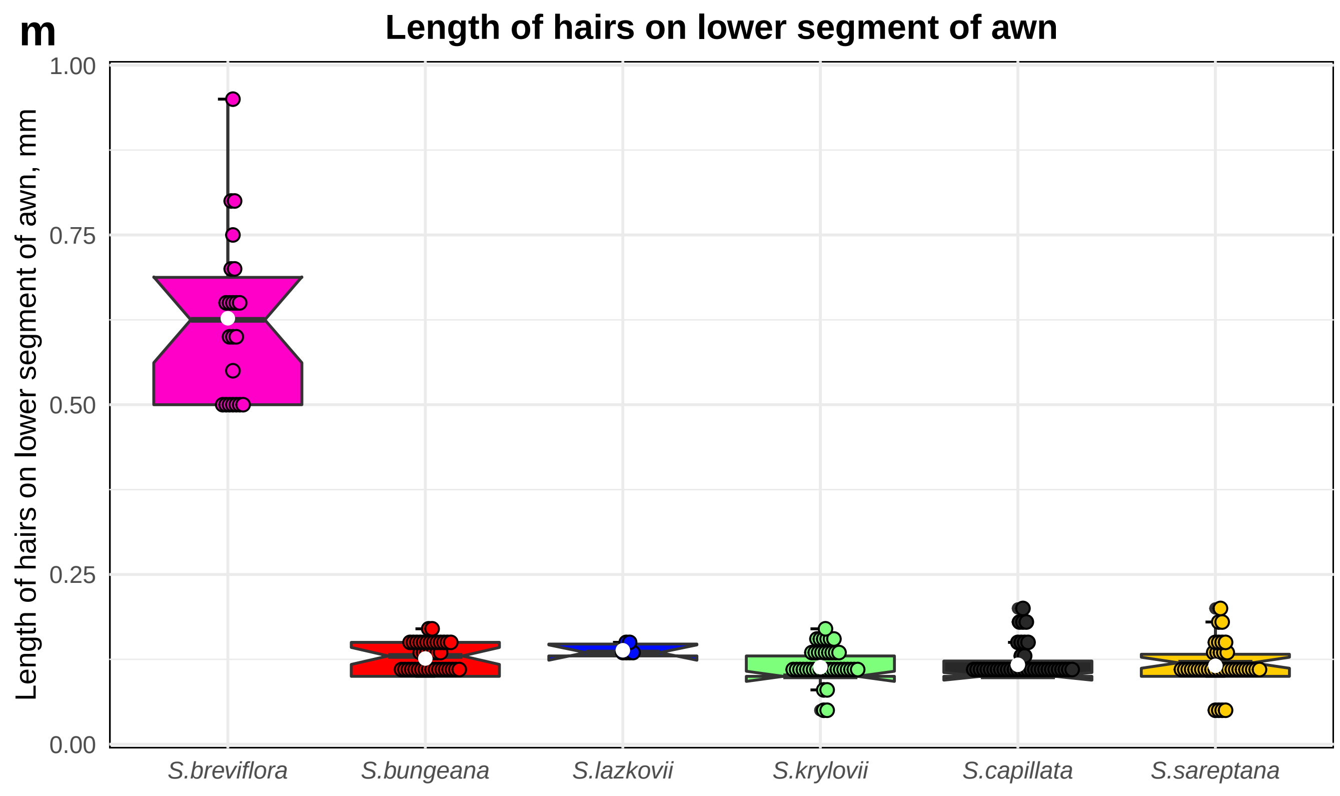


| | S.breviflora | S.bungeana | S.lazkovii | S.krylovii | S.capillata |
|---|---|---|---|---|---|
| S.bungeana | *** | | | | |
| S.lazkovii | ** | | | | |
| S.krylovii | *** | | | | |
| S.capillata | *** | | | | |
| S.sareptana | *** | | | | |

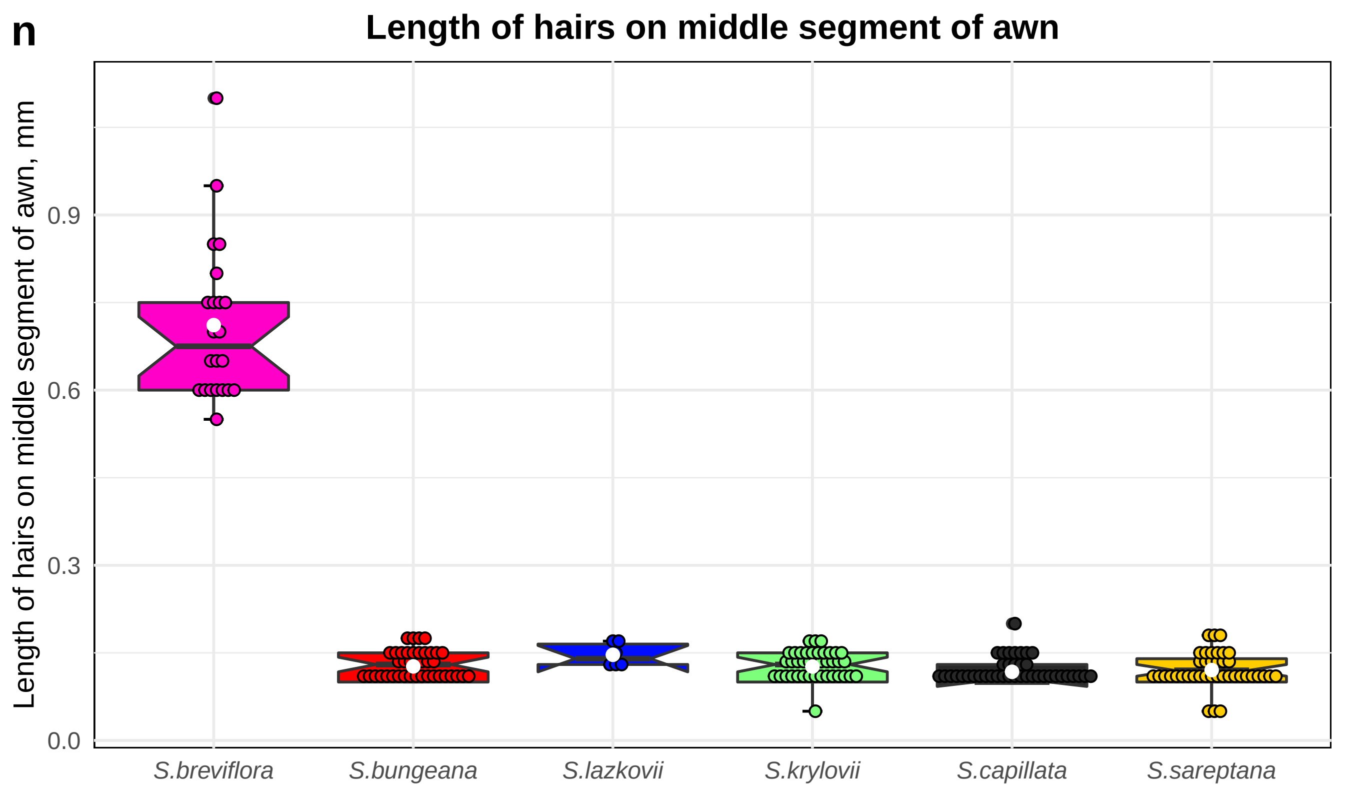


| | S.breviflora | S.bungeana | S.lazkovii | S.krylovii | S.capillata |
|---|---|---|---|---|---|
| S.bungeana | *** | | | | |
| S.lazkovii | ** | | | | |
| S.krylovii | *** | | | | |
| S.capillata | *** | | . | | |
| S.sareptana | *** | | | | |

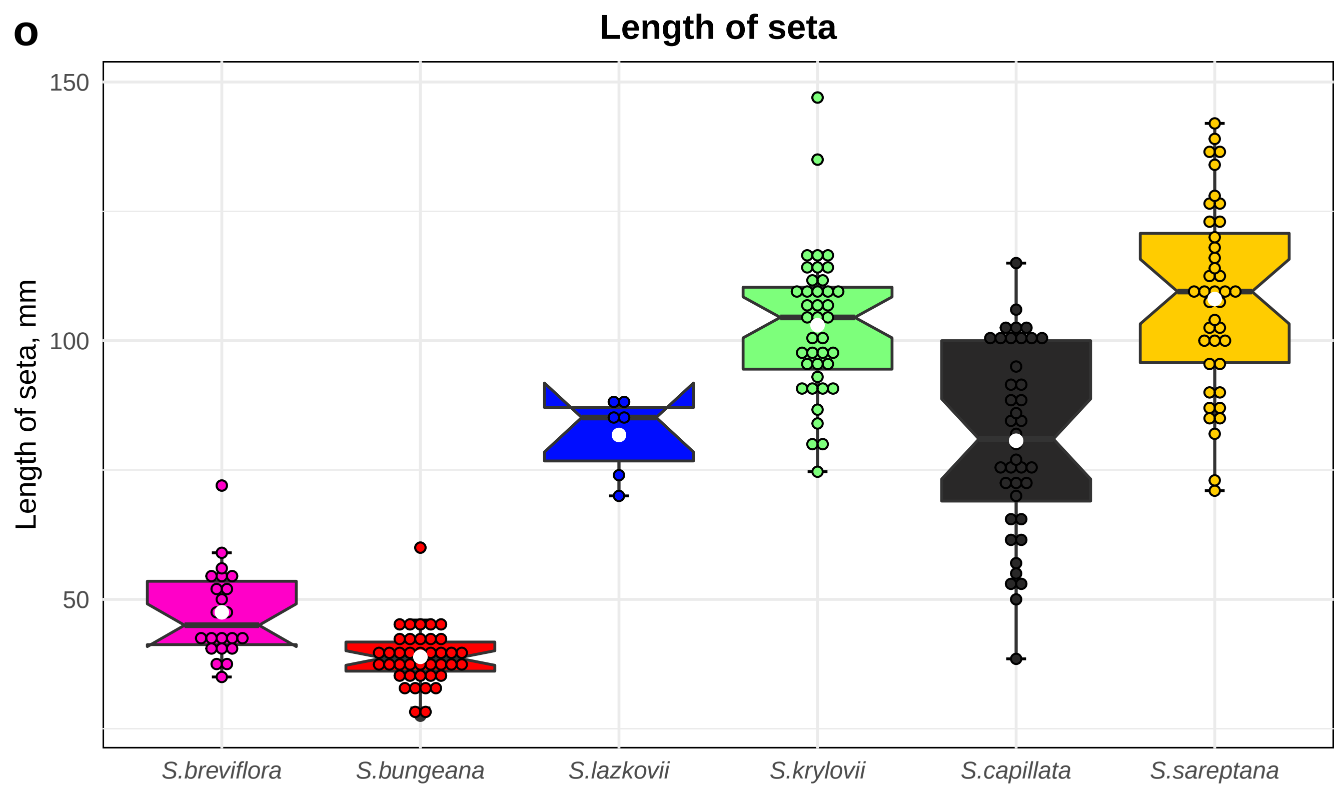


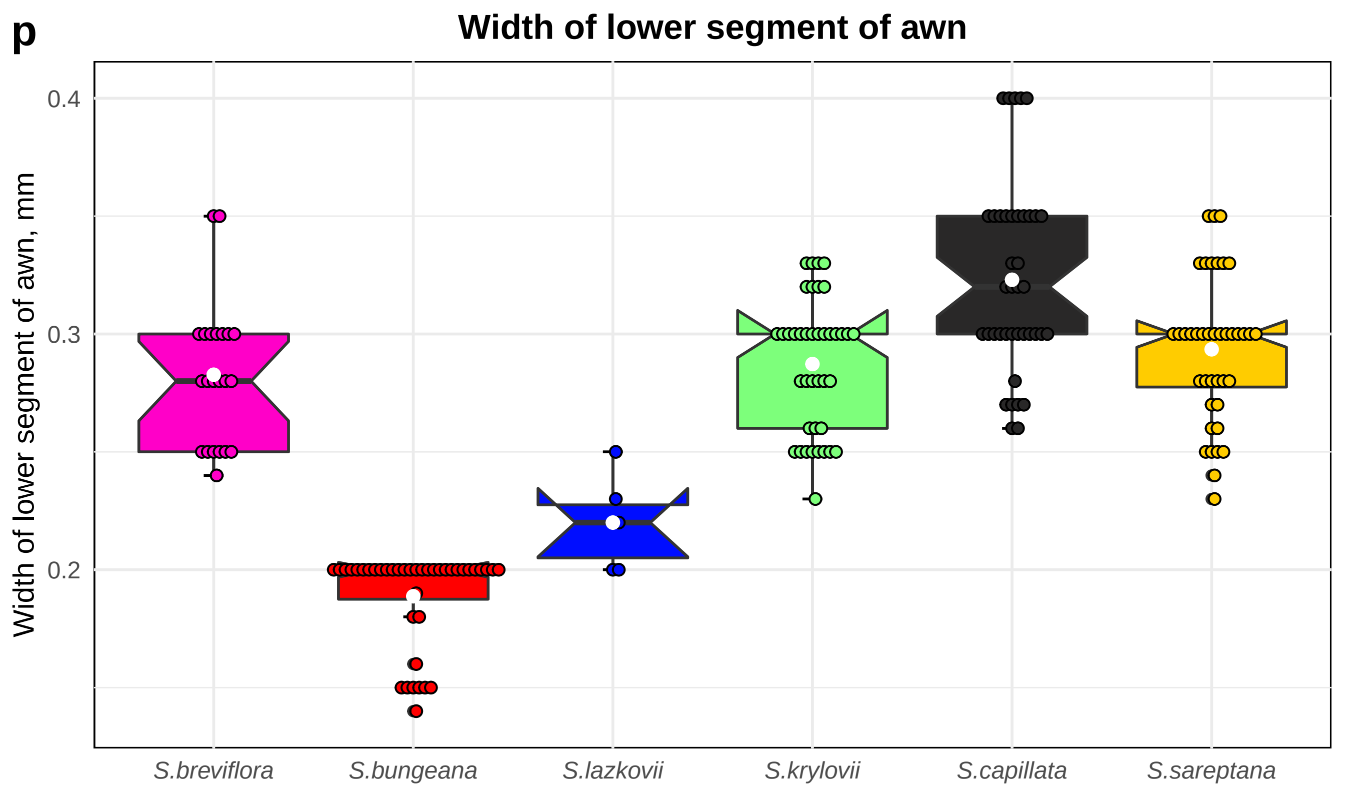


| | *S.breviflora* | *S.bungeana* | *S.lazkovii* | *S.krylovii* | *S.capillata* |
|---|---|---|---|---|---|
| *S.bungeana* | *** | | | | |
| *S.lazkovii* | ** | ** | | | |
| *S.krylovii* | *** | *** | * | | |
| *S.capillata* | *** | *** | | *** | |
| *S.sareptana* | *** | *** | * | | *** |

| | *S.breviflora* | *S.bungeana* | *S.lazkovii* | *S.krylovii* | *S.capillata* |
|---|---|---|---|---|---|
| *S.bungeana* | *** | | | | |
| *S.lazkovii* | ** | ** | | | |
| *S.krylovii* | | *** | ** | | |
| *S.capillata* | ** | *** | ** | ** | |
| *S.sareptana* | | *** | ** | | * |

q

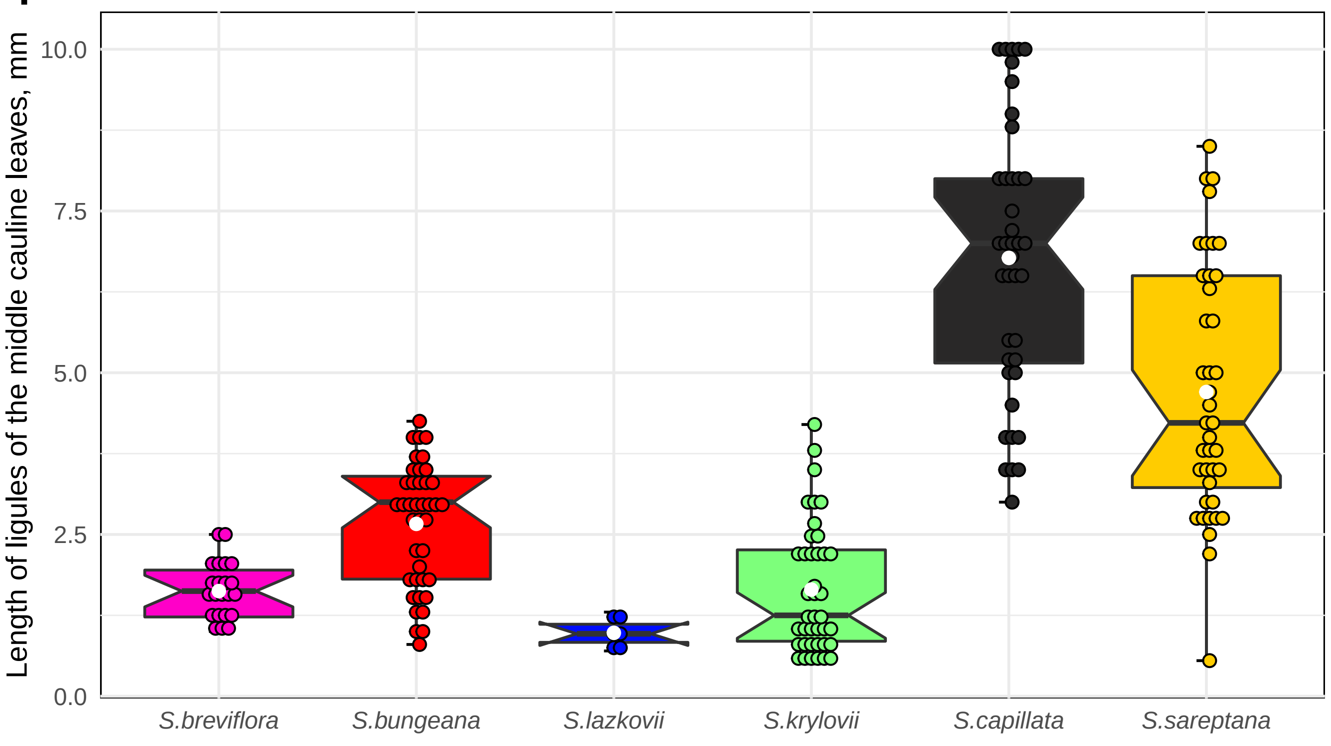


| | *S.breviflora* | *S.bungeana* | *S.lazkovii* | *S.krylovii* | *S.capillata* |
|---|---|---|---|---|---|
| *S.bungeana* | ** | | | | |
| *S.lazkovii* | * | ** | | | |
| *S.krylovii* | | *** | | | |
| *S.capillata* | *** | *** | ** | *** | |
| *S.sareptana* | *** | *** | ** | *** | *** |

r

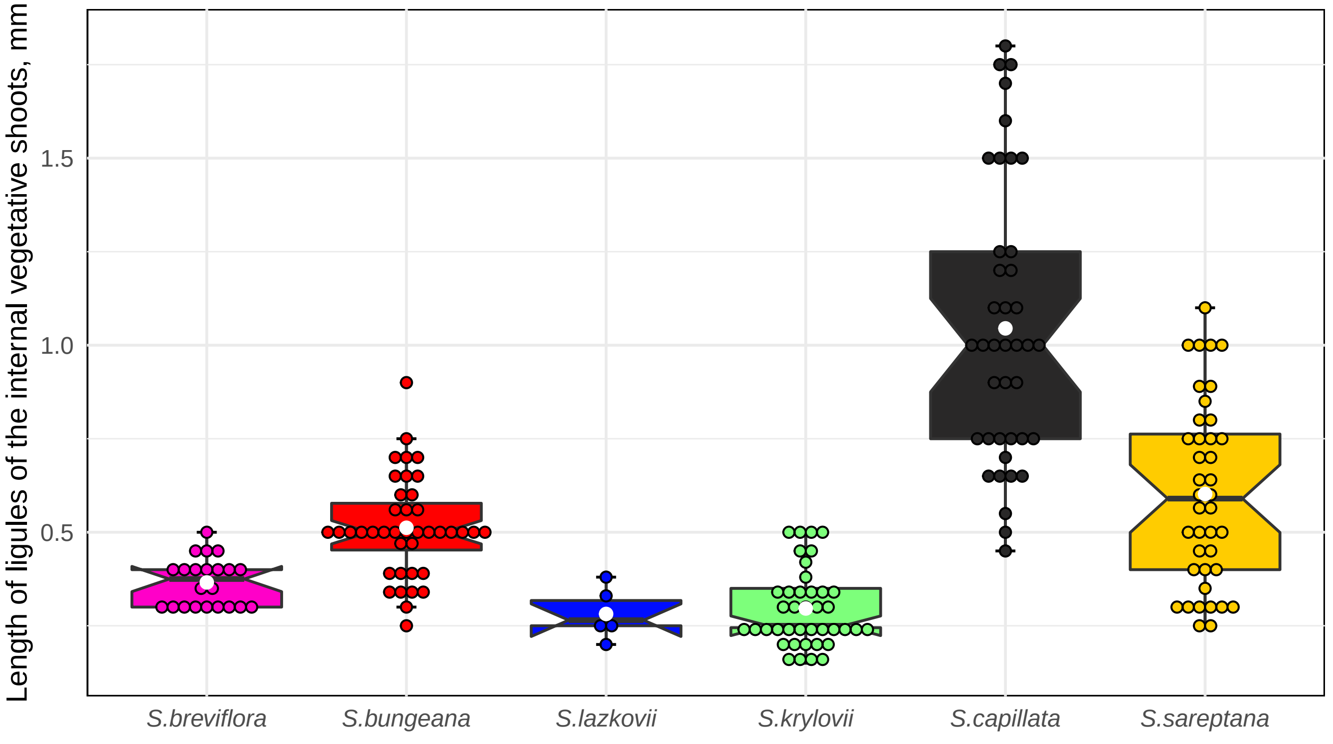


| | *S.breviflora* | *S.bungeana* | *S.lazkovii* | *S.krylovii* | *S.capillata* |
|---|---|---|---|---|---|
| *S.bungeana* | *** | | | | |
| *S.lazkovii* | | ** | | | |
| *S.krylovii* | * | *** | | | |
| *S.capillata* | *** | *** | ** | *** | |
| *S.sareptana* | ** | | * | *** | *** |

s

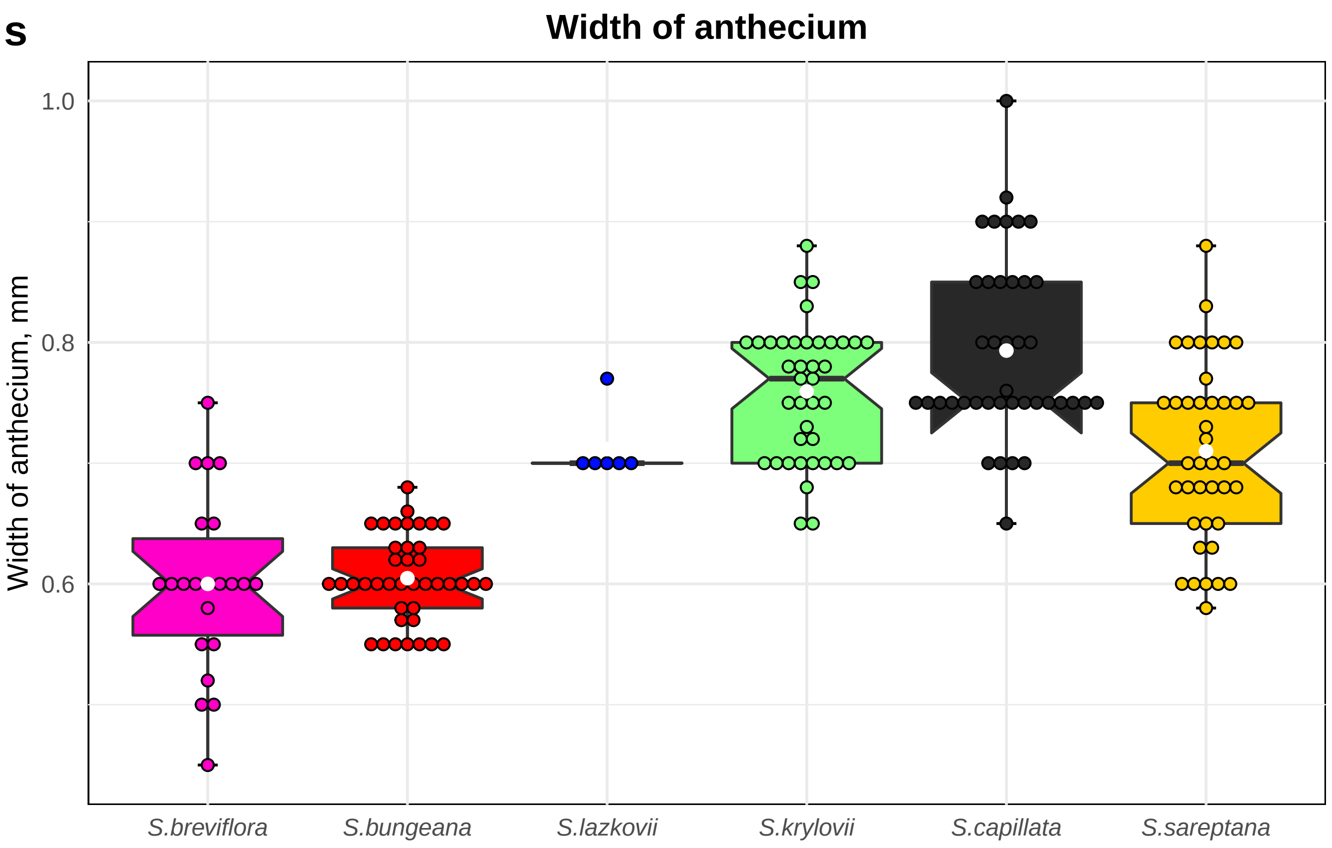


| | S.breviflora | S.bungeana | S.lazkovii | S.krylovii | S.capillata |
|---|---|---|---|---|---|
| S.bungeana | | | | | |
| S.lazkovii | * | ** | | | |
| S.krylovii | *** | *** | | | |
| S.capillata | *** | *** | . | | |
| S.sareptana | *** | *** | | * | *** |

t

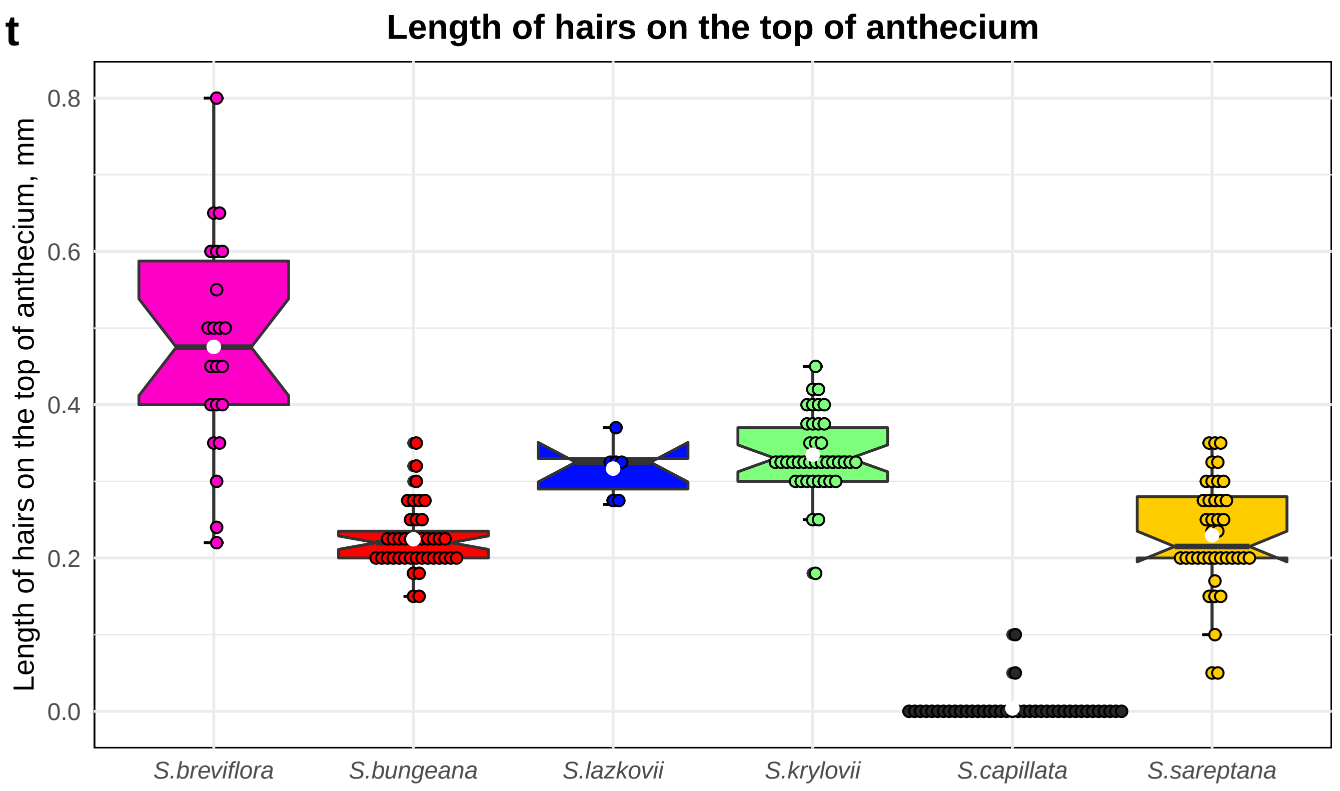


| | S.breviflora | S.bungeana | S.lazkovii | S.krylovii | S.capillata |
|---|---|---|---|---|---|
| S.bungeana | *** | | | | |
| S.lazkovii | | ** | | | |
| S.krylovii | *** | *** | | | |
| S.capillata | *** | *** | *** | *** | |
| S.sareptana | *** | | . | *** | *** |

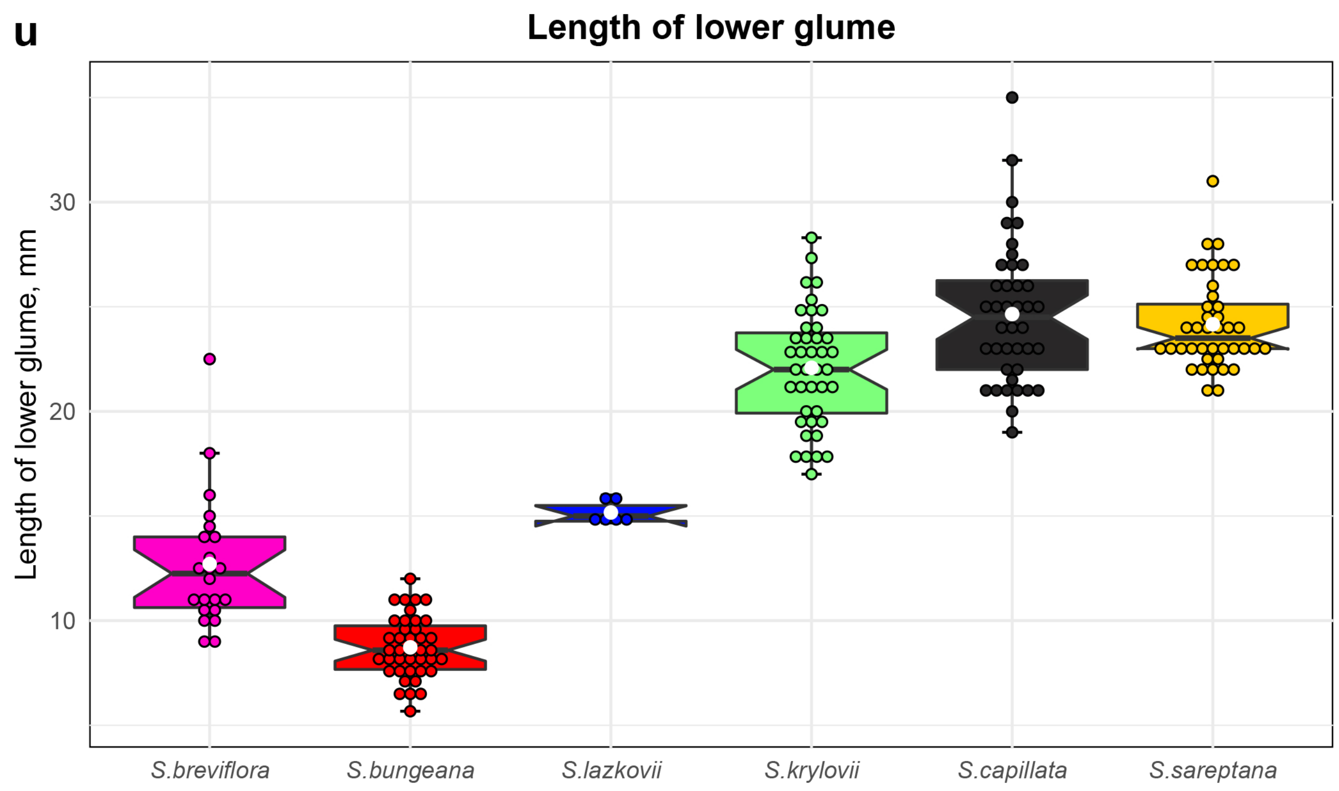


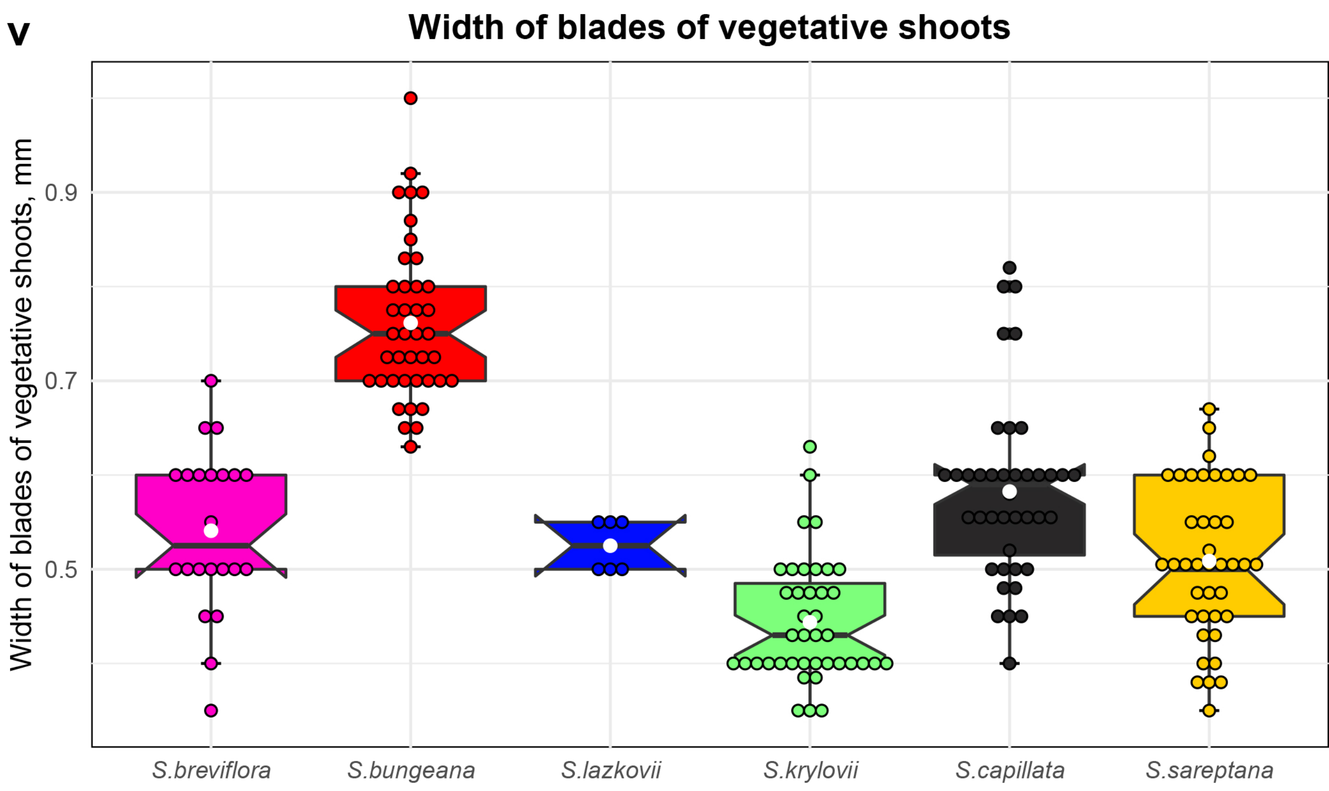


| | *S.breviflora* | *S.bungeana* | *S.lazkovii* | *S.krylovii* | *S.capillata* |
|---|---|---|---|---|---|
| *S.bungeana* | *** | | | | |
| *S.lazkovii* | | ** | | | |
| *S.krylovii* | *** | *** | ** | | |
| *S.capillata* | *** | *** | ** | * | |
| *S.sareptana* | *** | *** | ** | * | |

| | *S.breviflora* | *S.bungeana* | *S.lazkovii* | *S.krylovii* | *S.capillata* |
|---|---|---|---|---|---|
| *S.bungeana* | *** | | | | |
| *S.lazkovii* | | ** | | | |
| *S.krylovii* | *** | *** | * | | |
| *S.capillata* | | *** | | *** | |
| *S.sareptana* | | *** | | ** | * |

Supplementary Figure S1. Notched boxplot demonstrating the mean (white circle), the median (dark black line), 95% confidence interval around the median (notch), inter-quartile ranges (25% to 75%), whiskers (5% and 95%), and minimum and maximum measurements (crosses) of quantitative characters (a-v) for the studied species. Statistical significance was tested by Wilcoxon rank-sum test for post hoc group comparisons with Bonferroni correction, $p < 0.001$, $p < 0.01$, $p < 0.05$, $p < 0.1$, and $p < 1$ noted as '***', '**', '*', '.', and no symbol, respectively. Each dot represents an observation.

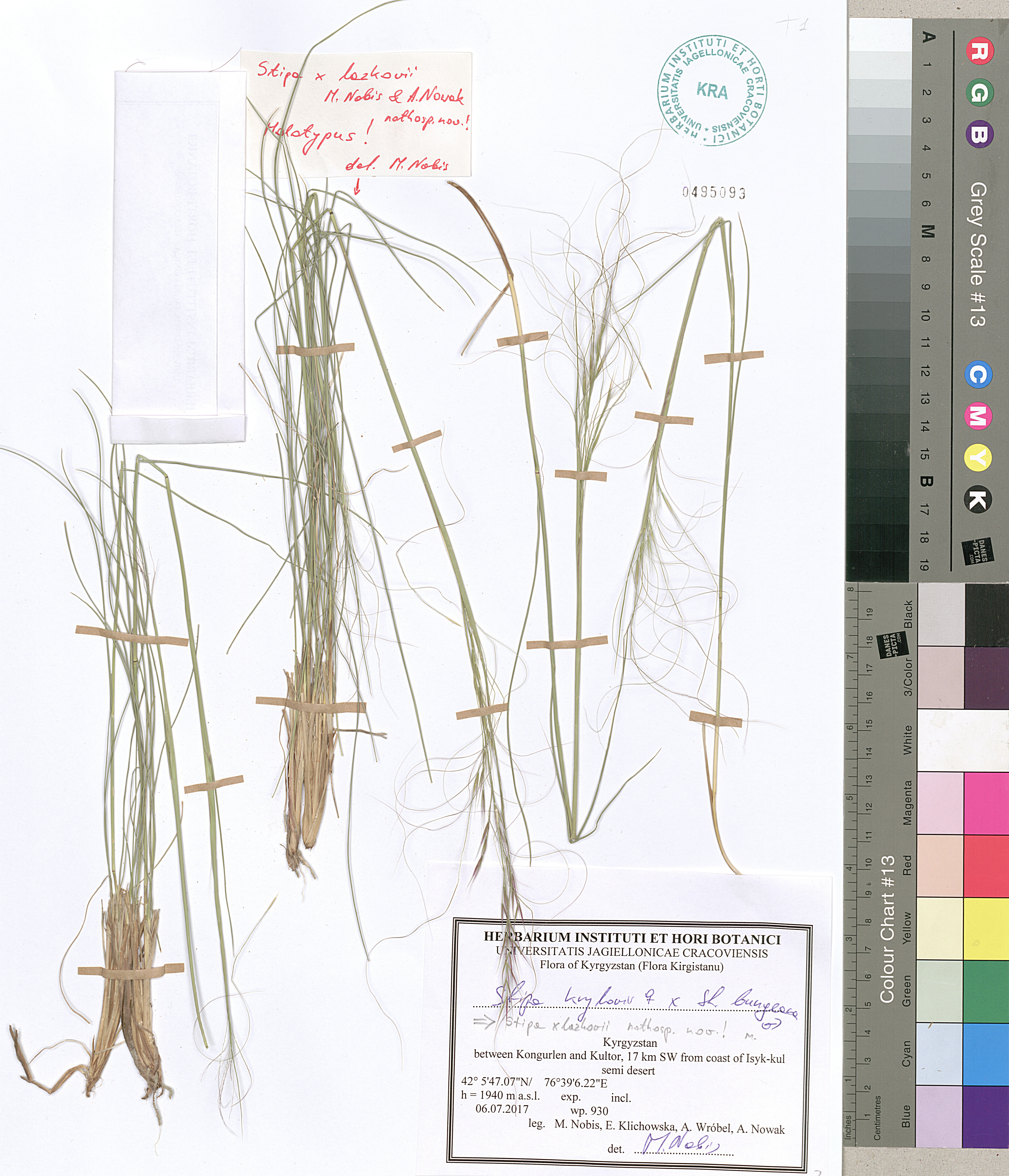


Supplementary Figure S2. The holotype of *Stipa ×lazkovii* M.Nobis & A. Nowak.

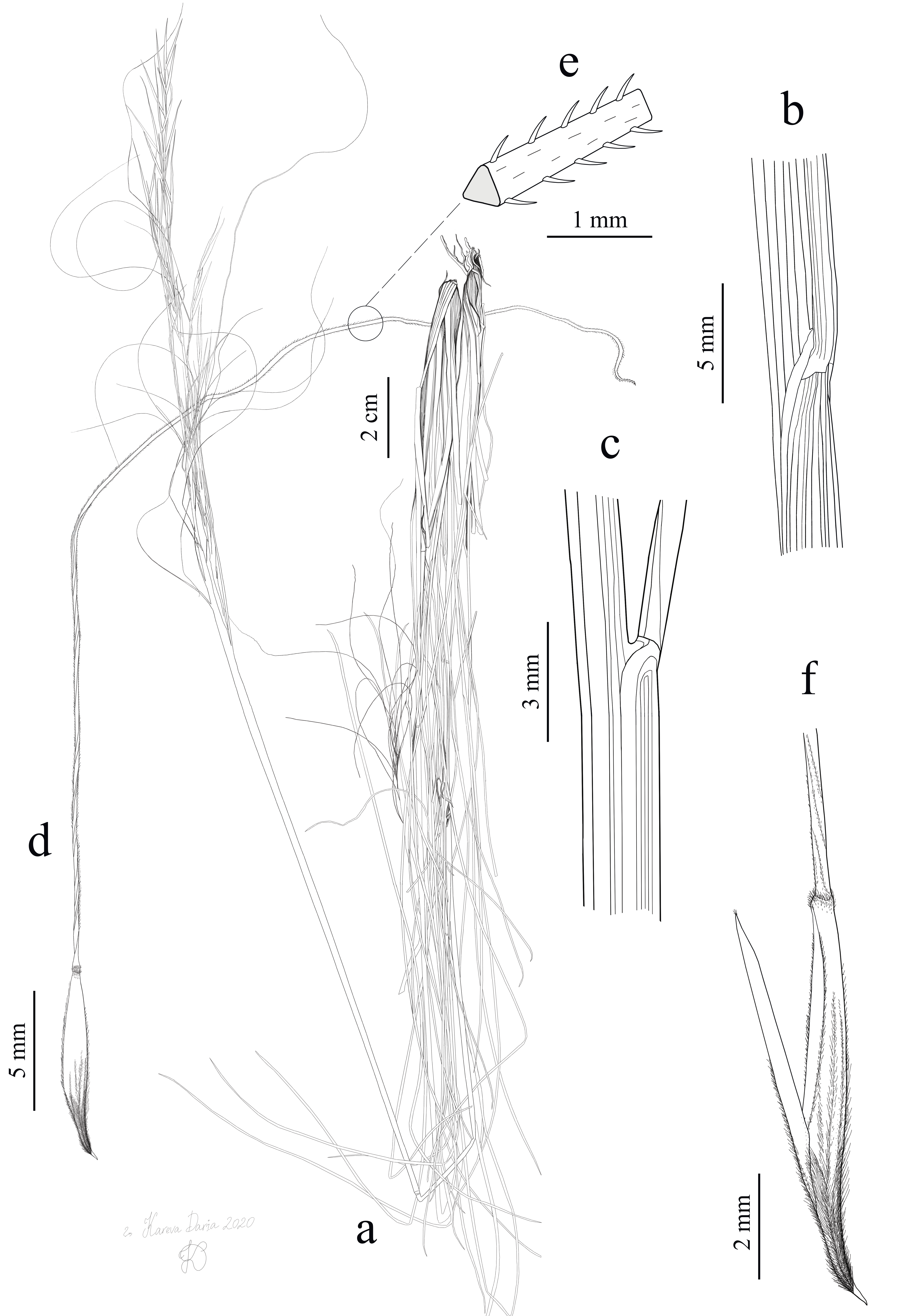


Supplementary Figure S3. The line drawing of *Stipa ×lazkovii*. (**a**) General habit. (**b**) Ligule of culm leaf. (**c**) Ligule of vegetative leaf. (**d**) Anthecium with awn. (**e**) The middle part of seta. (**f**) Anthecium.
